# Separating the effects of air and soil temperature on silver birch. Part I. Does soil temperature or resource competition determine the timing of root growth?

**DOI:** 10.1093/treephys/tpac092

**Published:** 2022-08-08

**Authors:** Jouni Kilpeläinen, Timo Domisch, Tarja Lehto, Sirpa Piirainen, Raimo Silvennoinen, Tapani Repo

**Affiliations:** Natural Resources Institute Finland (Luke), Yliopistokatu 6 B, 80100 Joensuu, Finland; Natural Resources Institute Finland (Luke), Yliopistokatu 6 B, 80100 Joensuu, Finland; School of Forest Sciences, University of Eastern Finland, Yliopistokatu 7, 80100 Joensuu, Finland; Natural Resources Institute Finland (Luke), Latokartanonkaari 9, 00790 Helsinki, Finland; Natural Resources Institute Finland (Luke), Yliopistokatu 6 B, 80100 Joensuu, Finland; Simitec Ltd., Nokkostie 2, 80160 Joensuu, Finland; Natural Resources Institute Finland (Luke), Yliopistokatu 6 B, 80100 Joensuu, Finland

**Keywords:** boreal tree species, fine root dynamics, growth allocation, root longevity, root morphology, root phenology, shoot phenology, soil warming

## Abstract

The aboveground parts of boreal forest trees mostly grow earlier, and the roots later, in the growing season. We aimed to experimentally test whether the extrinsic driver of soil temperature or the intrinsic driver (resource competition between plant parts) is a more important control for the root and shoot growth of silver birch (*Betula pendula* Roth) seedlings. Sixteen two-year-old seedlings were grown in controlled environment rooms for two simulated growing seasons (GS1, GS2). In GS1, all the seedlings were acclimatized under the same conditions, but in GS2, the soil temperature treatments were: (i) constant 10 °C (Cool); (ii) constant 18 °C (Warm); (iii) early growing season at 10 °C, switched to 18 °C later (Early Cool Late Warm, ECLW) and (iv) early growing season 18 °C, switched to 10 °C later (Early Warm Late Cool, EWLC). The treatments did not affect growth allocation between shoots and roots. Warm soil benefitted shoot elongation as it slowed down in EWLC and accelerated in ECLW after the soil temperature switch. However, whole-tree biomasses were similar to Cool and the seedlings grew largest in Warm. Phenology was not strongly affected by soil temperature, and root and shoot growth did not usually peak simultaneously. Short root mortality increased strongly in ECLW and decreased in EWLC after the soil temperature switch. Long root longevity was not significantly affected but long root growth ceased earliest in ECLW. Soil warming increased foliar nutrient contents. Growth dynamics were not solely driven by soil temperature, but resource competition also played a significant role. The study showed the importance of soil temperature for fine root dynamics not only through root growth but also via root mortality, as soil warming increased mortality even more than growth. Soil temperature has complex effects on tree and soil functioning, which further affects carbon dynamics in forest ecosystems that have a climate feedback.

## Introduction

The growth dynamics of boreal forest trees show strong seasonality, because the aboveground parts grow earlier in the growing season, and the roots mostly later (Abramoff and Finzi 2015). It is not well understood if the main reason for this is resource competition between above- and belowground parts, or if soil warming during the growing season increases root growth, both directly and via water and nutrient availability. Shoot and root growth and phenological events are driven by environmental factors, trees’ internal factors and their interactions (Radville et al. 2016*b*). The internal factors can be reduced to resource competition among plant tissues (or in other words, allocation among sinks and sources, depending on production) controlled by growth-regulator signaling, the phase of the annual cycle, ontogeny and heredity, but interaction with the environment remains. If the soil is sufficiently warm, stored assimilates can be used to initiate root growth in the spring, which continues in the autumn after aboveground growth has ceased (Pregitzer et al. 2000, Steinaker et al. 2010). Roots may not have a dormancy period like shoots, and roots and shoots can respond differently to changing environmental conditions (Radville et al. 2016*b*). Strong root and shoot expansion do not commonly occur simultaneously, which can be due to trade-offs among competing plant sinks (Lyr and Hoffmann 1967, Reich et al. 1980, Iivonen et al. 2001, McCormack et al. 2014). However, the relative importance of resource competition and the direct effects of soil temperature remain unclear, because aboveground growth is completed at about the same time as when the soil temperature has increased to annual maximum levels in cool and cold climates. The question here is whether root growth increases when the soil temperature is already high at the beginning of a growing season, or whether the resource competition favors shoot growth at the expense of root growth.

Soil temperature is one of the key factors affecting the functioning of trees and forest ecosystems. Different soil factors such as temperature and moisture are interconnected, but essentially, a low soil temperature during the growing season reduces root growth, as well as water and nutrient uptake, which affects the growth of aboveground parts, and according to feedback mechanisms, the growth of the roots (Pregitzer et al. 2000, Domisch et al. 2001, Yuan and Chen 2010). In temperate and boreal forests, root growth can begin in the spring before aboveground growth, but significant root growth is typically concentrated in the later part of the growing season (Steinaker and Wilson 2008, Steinaker et al. 2010, Ding et al. 2020). The early root growth suggests that root growth may not be limited by low soil temperature as such.

The functioning of soil microbiota is slow at low temperatures, thus also decreasing nutrient availability (Waksman and Gerretsen 1931, Biederbeck and Campbell 1973). A low temperature increases the viscosity of water and decreases the water permeability of cell membranes, which decline water uptake by the roots (Pallardy 2008). However, in the long run, trees may compensate for the lower nutrient availability in cold soil by growing more absorptive roots (Hertel and Schöling 2011, Ostonen et al. 2011, Zadworny et al. 2016). Apparently for the same reason, the proportion of growth allocation to roots tends to increase as we move towards colder climates with low soil temperature and fertility (Ostonen et al. 2007, Leppälammi-Kujansuu et al. 2014, Finér et al. 2019). In addition, fine roots generally have a longer lifespan in cold than warm soils (Leppälammi-Kujansuu et al. 2014, Kilpeläinen et al. 2019). This may be explained by increased root maintenance respiration in warm soils, which leads to earlier root senescence (Eissenstat et al. 2000). The longevity of roots should therefore also be taken into account when considering the timing of root growth.

The rooting zone temperature during the growing season in the boreal area ranges between 5 °C and 20 °C, with averages between 10 °C and 12 °C (Kubin and Kemppainen 1991, Domisch et al. 2001). The root growth of boreal tree species has been shown to be significantly lower at 10 °C than at 18 °C, the latter being closer to an optimum (Lyr and Hoffmann 1967, Domisch et al. 2001, Solfjeld and Johnsen 2006, Alvarez-Uria and Körner 2007). We therefore selected 10 °C and 18 °C for the soil temperature treatments in our experiment. It is difficult to study the drivers of root and shoot growth in field conditions, because the effect of soil temperature is confounded with air temperature. In addition, the soil water content may also be affected by soil temperature via altered evapotranspiration. These confounding effects can be separated in controlled laboratory conditions.

Here, we studied the effects of soil temperature in the control of root and shoot growth of silver birch (*Betula pendula* Roth) seedlings by switching the soil temperature crosswise between low and high during the growing season while keeping the other conditions constant. The main aim was to test the effects of soil temperature as the external driver of the growth dynamics of the shoots and roots. The aim was to separate these effects from the internal drivers by crosswise switching the soil temperature during the growing season from low to high (Early Cool—Late Warm, ECLW) and from high to low (Early Warm—Late Cool, EWLC), in addition to the treatments with constant cool and warm soils. Growth and the timing of the phenological events of roots and shoots, root morphology, mortality and longevity, foliar nutrients and soil gas concentrations were examined. Concurrent changes in soil conditions were investigated to gain a more comprehensive view of soil–plant interactions affected by soil temperature. We hypothesized that a high soil temperature in the latter part of the growing season, simulating boreal field conditions, would increase root growth. On the other hand, if soil temperature was the main driver of root growth instead of resource competition, a low soil temperature in the latter part would inhibit root growth.

## Materials and methods

### 2.1. Plant material and growing conditions

Silver birch (*Betula pendula* Roth) seedlings were raised in Massbybacka tree seedling nursery in southern Finland (Sipoo, 60°18′N 25°17′E; seeds from Tapio Siemenkeskus, seed lot T03-14-0006) in 1.3-l pots. In the autumn of 2017, two-year-old seedlings were stored in field conditions in Joensuu, eastern Finland (62°36.3′N 29°44.5′E, 80 m above sea level) to gain natural chilling and release bud dormancy before the start of the experiment at the end of November. The seedlings were planted in four dasotrons (RTR48, Conviron, Winnipeg, Man., Canada) in the Joensuu root laboratory with four containers (diameter 70 cm, height 59 cm) in each dasotron, and one seedling in each container (Finér et al. 2001). The containers were filled with a 7-cm layer of fine gravel at the bottom followed by a filter fabric, a 22-cm layer of fine sand (≤1 mm grain size), a 22-cm layer of fine mineral soil from a silver birch stand, and an 8-cm layer of topsoil from a silver birch stand. The type of mineral soil collected from the birch stand was fine sand till with a median grain size of 0.14 mm. The soil water content was ca. 27% near field capacity (matric potential ca. −10kPa, Heiskanen & Mäkitalo 2002) (Figure S1 available as Supplementary data at *Tree Physiology* Online). The lower cooling coil was 7 cm from the container bottom, and the upper coil on top of the soil organic layer. At planting, the soil and air temperatures were 3 °C, the photoperiod was short (6/18h day/night), the photon flux density was 100 μmol m^−2^ s^−1^ PAR from LED bars (BX-Series, Valoya Oy, Helsinki, Finland), and the relative air humidity was 90%.

**Figure 1.**
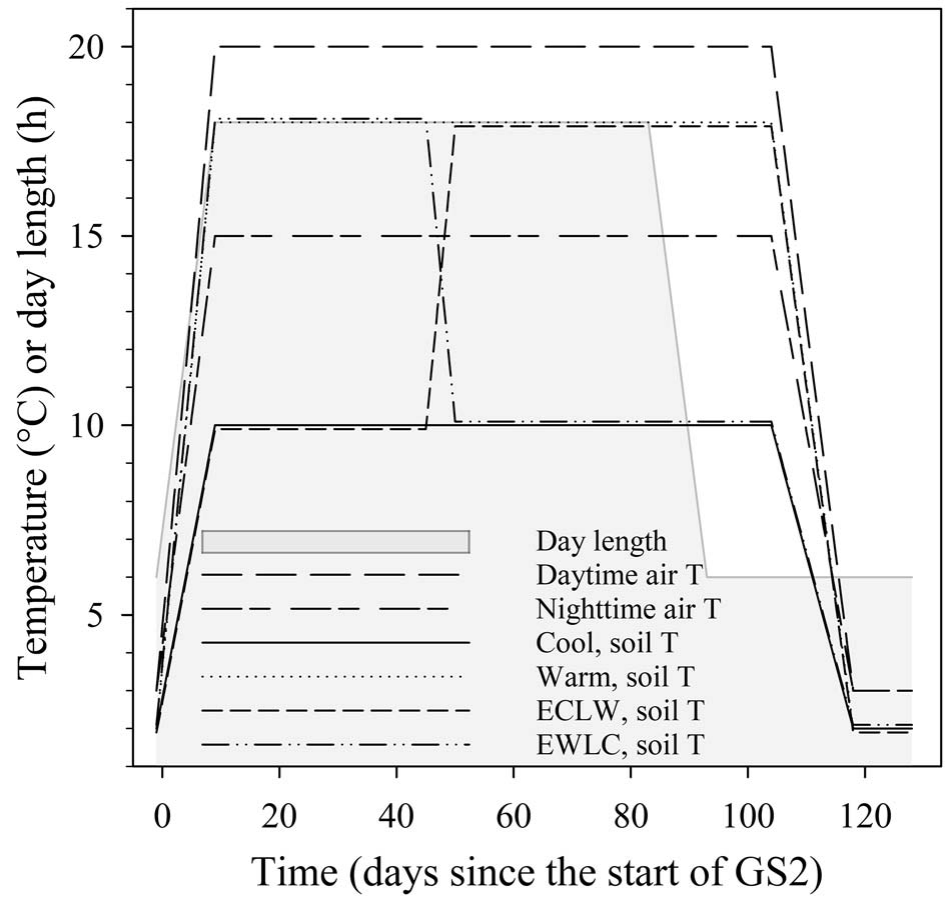
Illustration of the temperature treatments and day length in the second growing season of the silver birch experiment. For clarity, soil temperature is drawn 0.1 °C apart from the actual values in Early Cool & Late Warm (ECLW) and Early Warm & Late Cool (EWLC).

The seedlings were grown in the root laboratory for two simulated growing seasons (GS1 and GS2). GS1 was applied to acclimate the seedlings in the chamber conditions (Table 1), while the soil temperature treatments were carried out in GS2 (Figure 1). In GS1, there was an 11-week long-day phase (LD), and in GS2 a 12-week LD, and the LD was followed by a 3-week short-day phase (SD). A 10-week dormancy period (D1) was interposed between GSs. The conditions were changed gradually in ten days from D1 to GS2 and from LD to SD conditions, and in two weeks from SD to D conditions. The experiment ended in D2.

**Table 1:** Dasotron conditions during the experiment. GS stands for growing season, LD for long-day phase, SD for short-day phase, D for dormancy period, RH for relative air humidity and PAR for the photon flux density of photosynthetically active radiation. The soil temperature treatments were carried out during GS2.

	GS1/LD	GS1/SD	D1	GS2/LD	GS2/SD	D2
Time, days since GS2 started	−168	−91	−70	0	84	105
Phase duration, days	77	21	70	84	21	23
Air temperature (day/night), °C	20/15	20/15	3/3	20/15	20/15	3/3
RH (day/night), %	60/85	60/85	90/90	60/85	60/85	90/90
PAR, μmol m^−2^ s^−1^	400	100	100	400	100	100
Photoperiod (day/night), h	18/6	6/18	6/18	18/6	6/18	6/18
Soil temperature, °C	15	15	2	10 or 18	10 or 18	2

The soil temperature treatments in GS2 were: (i) constant 10 °C (Cool); (ii) constant 18 °C (Warm); (iii) early growing season at 10 °C, switched to 18 °C in the mid of growing season (Early Cool Late Warm, ECLW) and (iv) early growing season 18 °C, switched to 10 °C in the same phase as in ECLW (Early Warm Late Cool, EWLC) (Figure 1). Among the treatments, ECLW most resembles the natural conditions. The soil temperature was switched gradually in five days (46–50 days after the start of GS2). Hereafter, days refer to days after the start of a growing season, and the letters E and L with day numbers refer to early and late GS2 (before and after the soil temperature switch, respectively). The air temperature sum with a 5 °C threshold was ca. 1220 dd (degree-days) in GS1 and 1330 dd in GS2. The soil temperature sums, with a 5 °C threshold, were ca. 920 dd in GS1, and 495 dd, 1295 dd, 950 dd and 840 dd in Cool, Warm, ECLW and EWLC in GS2, respectively. The chilling unit (CU) accumulation in D1 was about 52 CUs (Hänninen 1990). The soil moisture was kept near field capacity, with irrigation twice a week during growing seasons and biweekly during dormancy periods. During a simulated year, a nutrient deposit representing that in annual rainfall in southern Finland (based on the averaged data of Finnish Meteorological Institute, Virolahti and Ähtäri stations, 2016, and the Finnish Environment Institute, Loviisa, Utti, Laukaa and Ylistaro stations, 1998) was given with irrigation water. In each watering, a fixed dose of 9.9 mg N, 0.18mg P, 0.97 mg K, 1.85 mg Ca, 4.95 mg Cl, 0.47 mg Mg, 2.9 mg Na and 2.9 mg S was given for each seedling.

### 2.2. Soil characteristics, plant dry matter and nutrients

The dry mass content (measured by drying at 105 °C), loss on ignition (LOI, measured by burning at 550 °C) and pH (1:2 in deionized water) were determined for samples of soil organic layer and mineral soil pooled from the containers of each dasotron at the start of the experiment. At the start, LOI was 13.6 ± 0.28% (mean ± SE, *n* = 4) of dry matter (105 °C) in the soil organic layer and 1.85 ± 0.02% (mean ± SE, *n* = 4) in the mineral soil layer. The pH of the soil organic layer was 5.34 ± 0.09, and that of the mineral soil was 5.36 ± 0.02. At the beginning of the experiment, the mineral soil included < 0.8 mg g^−1^ N and exchangeable and easily soluble K 9.1, P 1.2, Ca 17.1, Fe 7.4, Mg 1.7, Mn 3.9 and S 30.3 μg g^−1^. At the start, the total content of N, P and K was 1.88 mg g^−1^, 588 μg g^−1^ and 813 μg g^−1^ in the soil organic layer, respectively (Table S1 available as Supplementary data at *Tree Physiology* Online). At the end of the experiment, separate soil samples were taken from the soil organic layer and mineral soil of each container for LOI and pH analyses.

Silver birch has two leaf flushes; i.e., short-shoot leaves emerge from overwintered buds in the spring, and long-shoot leaves from the new buds of the current growing season. Two to three short-shoot leaves were sampled for carbon (C) and nitrogen (N) analysis from the upper third of the canopy of each seedling at E44 and L79 days after the start of GS2, and 10 long-shoot leaves for analysis of C and N and other nutrients at L81 days after the start of GS2. The short-shoot leaves were freeze-dried (Christ Alpha 1–4 LD, Martin Christ Gefriertrocknungsanlagen GmbH, Osterode, Germany) and ground with a ball mill (Fritsch Pulverisette 23, Fritsch GmbH, Idar-Oberstein, Germany) before analysis with a TruMac CN analyzer (Leco Co., St. Joseph, MI, USA). The long-shoot leaves were dried at 40 °C, ground with the mill and analyzed for C and N with the CN analyzer, and for other nutrients with ICP-OES (Thermo Scientific iCap 6500 Duo, Thermo Fischer Scientific, Waltham, MA, USA) after MARS5 microwave wet digestion in HNO_3_ and H_2_O_2_ in Teflon containers (method based on Epa 3051). The short-shoot leaves were also used for analyses of non-structural carbohydrates (Kilpeläinen et al., in press), and the freeze-drying was part of this analysis protocol.

Fallen leaves were collected regularly during GS2 and D2. The stems and branches of the previous and latest growing seasons, as well as the leaves and roots, were separated in the final harvest during D2. The current stems and branches included only the new stem elongation, not the new volume growth of the old stems and branches. The plant parts were dried at 40 °C and weighed. Foliar nutrient amounts were estimated for each seedling by multiplying the mean nutrient content of short- and long-shoot leaves by the dry mass of short- and long-shoot leaves and fallen leaves at the end of the experiment.

Oxygen (O_2_) concentrations (Fibox 4 trace, PreSens, Regensburg, Germany) were measured at a depth of 10 cm in mineral soil at 2 h intervals. Daily averages were calculated, and the same days as in the other soil gases were selected for statistical testing. Carbon dioxide (CO_2_), methane (CH_4_) and nitrous oxide (N_2_O) concentrations were determined at 2–4-week intervals during GS2, using silicone tubes (length 1 m and inner diameter 1 cm), the first tube set between the soil organic layer and mineral soil, and the second between the mineral soil and the fine sand layer. From the tubes, 40–50 mL of air was sucked into plastic syringes, transferred into 6-ml glass vials with a needle (Chromacol®, Sun Sri, Rockwood, TN, USA, caps: BUTYL liner, spring and crimp) and analyzed on the same day using a gas chromatograph (TurboMatrix and Clarus 580 GC, PerkinElmer, Waltham, MA, USA) equipped with a PlotQ capillary column and a two-channel flame ionization detector (FID). Ethylene (C_2_H_4_) effluxes from the soil were measured with a portable C_2_H_4_ analyzer (CI-900, BioSciences, Camas, WA, USA) at 2–4-week intervals during GS2. An opaque soil chamber (area 43.4 cm^2^, volume 0.634 dm^3^) was set on the soil surface, and changes in C_2_H_4_ concentrations (ppm) were recorded for 6–8 min. Ethylene effluxes (*F_A_*, g C_2_H_4_ m^−2^ h^−1^) were calculated using the following equation:

\begin{equation*} {F}_A=\frac{dC}{dt}\times \frac{P\times M\times V}{R\times A\times T}, \end{equation*}

where *dC*/*dt* (mol mol^−1^ h^−1^) is the C_2_H_4_ accumulation rate inside the chamber, calculated from the linear regression of C_2_H_4_ concentration against time (*t*), *P* is the atmospheric pressure (101,325 Pa), *T* is the chamber temperature (K), *V* is the chamber volume (m^3^), *A* is the area of the chamber base (m^2^), *M* is the molecular mass of C_2_H_4_ (28.05 g mol^−1^) and *R* is the gas constant (8.314 JK^−1^ mol^−1^).

### 2.3. Shoot and root growth and phenology

The budburst of the top apical bud of the main shoot and five axillary buds below it were monitored at the beginning of the growing seasons. The stem diameter and shoot height were measured at 5–9-day intervals during GS1 and GS2. The initiation day of stem diameter growth was determined as the midpoint of the imaging session, where the diameter increment reached 5% of the total diameter increment of the growing season and its preceding imaging session. Shoot elongation was initiated at budburst. The days of maximum rates of diameter and height growths were the midpoints of the measuring intervals that included the maximal growth rates. The cessation days of stem diameter and height growths were the midpoints of the measuring interval when the maximum values were reached. Shoot height and diameter increments were proportioned to their values at the start of the growing season.

The roots were digitally photographed (Bartz BTC-100X Camera System, Bartz Technology Co., Santa Barbara, CA, USA) through the upper side of an acrylic minirhizotron tube, 60 mm in outer diameter, installed horizontally in each pot, with the upper side 15–16 cm below the soil surface. In each tube, 46 frames of 13 × 18mm^2^ were photographed at ca. 3-week intervals. In the image analysis, the RootView software (Aphalo and Simonic 1999) was used to assess the appearance, length, death and disappearance of the roots. First-order roots without branching were defined as short roots, and the higher-order roots as long roots. If a particular root changed from the first to a higher order during the study, it was changed into a long root retrospectively for all imaging sessions. Extensive ectomycorrhizal colonization was visible in the minirhizotron images, but the colonization rate was not determined. Most short roots were most probably ectomycorrhizal because based on a field study more than 95% of silver birch short roots can form ectomycorrhizas (Uri et al. 2007). These short roots function as absorptive roots while long roots function as transport and pioneer roots (Freschet et al. 2021). A root was considered dead when it started to appear disintegrated in the image, and to be disappeared when it was no longer visible. The visual determination of root death lags the actual time of death (Wojciechowska et al. 2020) but still gives a reasonable estimate for root mortality assessed by interval imaging.

The standing root length per unit image area (*l*_area_, m m^−2^) was calculated by dividing the total length of live roots by the area of the image frames. The net increment of *l*_area_ (m m^−2^) from a GS start was calculated by subtracting the *l*_area_ at the beginning of a GS from the *l*_area_ at each later imaging session. The net root elongation rate per unit image area (Δ*l*_area_, m m^−2^ d^−1^) was the difference in *l*_area_ between two consecutive imaging sessions divided by the number of days (d) in the interval. Both new root appearance and old root elongation were included in Δ*l*_area_. Root mortality per unit image area (m m^−2^) was calculated by dividing the length of roots that died between two consecutive imaging sessions by the area of the image frames, and for root mortality rate (m m^−2^ d^−1^) further divided by the number of days. Because the absolute root mortality depended on *l*_area_, the mortality share was also calculated as the ratio of dead root length to the sum of live root length, and the cumulative dead root length at each imaging session for GS1 and GS2. Additionally, the temporal patterns of root growth (viz. distributed pattern, unimodal pattern or bimodal pattern with a dominant growth peak in the spring or autumn, or equal peaks) as described by McCormack et al. (2014) were visually assessed from the figures. All parameters were calculated separately for short and long roots.

The day of root growth initiation (the appearance of new roots and/or the elongation of existing roots) was determined as the midpoint of the imaging session, where Δ*l*_area_ reached 5% of the sum of the positive Δ*l*_area_ values of the growing season and its preceding imaging session. Because some seedlings also grew some roots continuously during the dormancy period, the 5%-threshold value was selected to indicate the time of the start of significant root growth. The day of maximum root standing length was the day at the end of the interval, when *l*_area_ reached its maximum during a growing season. The day of the maximum rate of root growth was determined as the midpoint of the imaging interval when the daily Δ*l*_area_ reached its maximum during the growing season. The day of root growth cessation was determined as the midpoint of the latter imaging interval when the Δ*l*_area_ of two consecutive imaging intervals was less than 5% of the sum of the positive Δ*l*_area_ values of the growing season (cf. Radville et al. 2016*a*, Kilpeläinen et al. 2019). The time difference between the peaks in root and shoot growth rates was calculated as the difference in days when the maximum shoot and root growth rates were reached (equivalent to the offset described by Abramoff and Finzi (2015)).

Fine root turnover (FRT) reveals how many times the fine root population is computationally replaced during a period (here, a year). It was estimated separately for short and long roots, using two different methods: (i) the inverse of median root longevity (FRT_inv_med_) and (ii) the inverse mean root longevity (see survival analysis below) (FRT_inv_mean_) (Majdi et al. 2005). The FRT_inv_med_ and FRT_inv_mean_ were based on actual root longevity estimates during the whole study period (the unit is actual year^−1^).

### 2.4. Root morphology

At the final harvest, a 385-cm^2^ soil sector of the top organic layer (depth of 8 cm) and three 10-cm-thick mineral soil layers (to a depth of 38 cm) were harvested from each container for root morphology and biomass analyses. The roots were separated from the sectors, scanned at 400 dpi (Epson Perfection V750; Seiko Epson Co., Suwa, Japan), and their length, surface area, and volume were assessed with WinRHIZO (v. 3.1.2, Regent Instruments, Quebec, Que., Canada). The program calculates results for different diameter classes, here for four diameter (d) categories (*d* ≤ 0.5 mm, 0.5 < *d* ≤ 1 mm, 1 < *d* ≤ 2 mm and 2 < *d* ≤ 4.5 mm). The roots were dried at 40 °C until constant mass and weighed. Root morphology and biomass were upscaled for the whole root system in the 38-cm soil layer considering the sector sample volume. The rest of the root system, including the fine and coarse roots and stump up to the topmost root collar, was washed with water and assessed for dry mass as above. Their biomass was added to the extrapolated root biomass to calculate the total root biomass (DM_tot_). Specific root length (SRL) was calculated as the ratio of the scanned root length to the measured dry mass of the roots for categories *d* ≤ 2 mm and 2 < *d* ≤ 4.5 mm.

### 2.5. Electrical impedance spectroscopy of roots

The complex electrical impedance spectra (EIS; defined as the frequency response of the real and imaginary parts) of the root systems and stems were measured at 36 frequencies of an alternating current between 90 Hz and 200 kHz, with an impedance analyzer (EIS101, Simitec Ltd, Joensuu, Finland). The EIS as a non-destructive biophysical method provides information on the electrolyte balance of the plant cells, and changes in this balance will result in changes in the proportion of the alternating current passage along different routes including apoplastic and symplastic pathways within the plant sample (Repo et al. 2012, Jócsák et al. 2019). The method has been applied for instance in assessing frost damage of tree seedlings, but it can reveal differences also in plant samples treated more mildly (Di et al. 2019). The input peak-to-peak voltage level was 200 mV in the EIS measurement. Complex impedance is obtained from the relationship between voltage and current (regarding both amplitude and phase). Resistance forms the real part (*Z*_Re_), and reactance the imaginary part (*Z*_Im_), of the complex impedance (*Z*), the magnitude of *Z* being |*Z*|=$\sqrt{{Z_{\mathrm{Re}}}^2+{Z_{\mathrm{Im}}}^2}$, and the phase angle (i.e., loss factor) *δ* = tan^−1^ (*Z*_Im_/*Z*_Re_). Electrical impedance spectra were measured twice for each soil-root-stem continuum (below in this matter denoted roots). The measurement configuration included a stainless steel rod electrode (diameter 4 mm, length 300 mm) inserted into the soil 30 cm apart from the stem to a depth of 20 cm and stainless-steel needles (diameter = 1.5 mm) inserted to a depth of 2 mm at the opposite sides of the stem at 5 cm above the soil surface. To measure the stem, the soil electrode was disconnected, and another pair of electrodes was connected 5 cm above the lower stem electrodes. The measurements were repeated twice, with the stem electrodes set perpendicularly between the measurements. Thus, eight EIS were measured in each treatment for both roots and stems.

### 2.6. Root hydraulic conductance

Reverse-flow root hydraulic conductance (*K*_r_, g MPa^−1^ s^−1^) was measured at final harvest with a high-pressure flow meter (HPFM) (Dynamax, Houston, TX, USA). The stem was cut at 5 cm above the root collar. The bark was removed 3 cm below the top of the stump, and the capillary tube of the HPFM was joined to the cut surface. The measurement is based on monitoring the water flow by gradually increasing the pressure on the roots to 0.55 MPa (Tyree et al. 1995). *K*_r_ was obtained from the linear part of the relation between water flux and applied pressure. Temperature correction was calculated using the equation provided by the manufacturer (Dynamax 2020) as: *K*_r_ = *K*_raw_  ^*^ (0.554 + 0.0225 ^*^ T)/(0.554 + 0.0225 ^*^ T_c_), where *K*_raw_ is the measured conductance, T is the measurement temperature (here, soil temperature 2 °C), and T_c_ is the calibration temperature (here 22 °C). Here, the correction decreased the *K*_raw_ by ca. 43%.

### 2.7. Statistical analyses

The phenology results were tested with the one-way ANOVA, with treatment as the independent factor. The *l*_area_ increments of roots, root mortality, stem diameter and height and the share of dead root length were analyzed separately for GS1 and GS2 with a linear mixed model using the seedling as a subject variable and the day of the growing season as a repeated variable (covariance type AR1). The fixed effects model included soil temperature treatment, the day of the growing season and their interaction. Additionally, for *l*_area_, the values at the start of growing seasons were included as a covariate, and *l*_area_ was a covariate for root mortality. The random effects model included seedlings. Prior to the analyses, logarithmic (ln) transformations were applied for *l*_area_ and root mortality, and logit transformations were applied for the shares of dead root length. The foliar C and N content of short-shoot leaves, as well as soil gases, was tested with linear mixed models, in which the seedling was a subject, and the sampling time a repeated variable (covariance type AR1), and the soil temperature treatment, sampling time, and their interaction were fixed factors, while the seedling was a random factor. The one-way ANOVA was applied for the other nutrient contents, for all the nutrients of long-shoot leaves and all the nutrient amounts. The one-way ANOVA was also used for the loss factor at a frequency of 2.5 kHz. This gave the best resolution between treatments and for root morphology, HPFM and biomasses. The loss in ignition and the pH of the soil were tested for treatment (between-subject factor), and the soil layer (within-subject factor) differences with a repeated measures ANOVA. The offset was tested with a two-way ANOVA, with treatment, growing season and their interaction as factors. In the results, the significance probabilities are denoted with the subscripts *T* for temperature treatment, *t* for sampling time and adj. For adjusted pairwise comparison. If the main effects or interactions were significant (*P* ≤ 0.05) or nearly significant (0.05 < *P* ≤ 0.1) in the models, the Bonferroni correction post-hoc test was applied for pairwise comparisons. These analyses were run using IBM SPSS Statistics 26.0.0.0 software. Only one soil temperature, 15 °C, prevailed in GS1, but the tests were run as if all four treatments were already present. The not yet actualized treatments in GS1 are quoted for clarity (‘treatment’).

The EIS data were subjected to class-featuring information compression (CLAFIC) analysis as a four-class problem (Jaaskelainen et al. 1994, Repo et al., 2014*a*, Repo et al., 2016, Di et al. 2019, Repo et al. 2020). CLAFIC analysis is based on the principle of artificial intelligence, in which the training data (all measured spectra) are compared with each of the learning datasets. The classes (i.e., the learning data) were formed from the spectra of the soil temperature treatments. The measure of how the spectra in the classification groups resembled each other was calculated by comparing the number of spectra belonging to each group. In the classification, the unknown spectrum at a frequency range of 150 Hz–150 kHz was classified by measuring the length of the projection vector in each subspace *k*, where *k* took the fine structure of the spectrum into account (*k* = 1 represents approximately the mean value of an EIS set).

The roots appeared and died or disappeared during certain time intervals, based on the minirhizotron images taken at different times, and time to the event (i.e., root death) was within an interval in these interval-censored data. The nonparametric maximum likelihood estimation (NPMLE) of the survival function was carried out for short and long roots, and survival was compared among different soil-temperature treatments with an asymptotic logrank *k*-sample test (permutation form, Sun’s scores) using R version 4.0.4 and the interval package version 1.1–0.7 (Fay and Shaw 2010) through RStudio version 1.4.1106 (RStudio, Boston, Mass., USA). In right-censored point data, the NPMLE (called the Kaplan–Meier estimator) is undefined after the largest right-censored observation, because the NPMLE is not unique. In interval-censored data, the estimate is undefined because of non-uniqueness at certain intervals, and the survival curves plotted with the interval package are shown as descending slopes in these cases or as step functions, if they are uniquely defined (Fay and Shaw 2010). Survival analysis was run with data covering the whole study period to find out how the treatments affected both existing roots grown at similar conditions and roots produced during the treatments. For comparison, we run the analysis separately for cohorts including roots that appeared before the start of GS2, roots that appeared during or after GS2, and roots that appeared after the time of the soil temperature switch. Mean and median longevities and their confidence intervals were estimated based on the data covering the whole study period for short and long roots as done by Repo et al. (2014*b*). The median estimate was the first event time when the NPLME survival curve went below 0.5, and the mean estimate was the integral of the survival curve. The descending slope of the NPMLE curve was treated as an ad-hoc estimate of survival in relation to time during the intervals when it was undefined. The 95% confidence intervals were based on bootstrapping with 3000 resamples.

## Results

### 3.1. Root phenology

The soil temperature treatments did not show clearly in the initiation, cessation and maximum of *l*_area_, and in the maximum of Δ*l*_area_ of short and long roots. More specifically, there was large variation in the initiation of short root growth in GS2, and it did not differ significantly between treatments (*P*_T_ = 0.687) (Table 2). Nor did the initiation time for long root growth show treatment differences (*P*_T_ = 0.570) (Table 2). Only Cool and Warm were present at the time of root growth initiation.

**Table 2:** The times (in days after the start of the second growing season, GS2) of initiation and cessation of short and long root elongation, and maximum root length (*l*_area_) and maximum rate of root elongation rate (Δ*l*_area_) in silver birch seedlings in different soil temperature treatments during GS2 (*n* = 4) (Cool = constant 10  °C, Warm = constant 18  °C, ECLW = Early Cool & Late Warm, EWLC = Early Warm & Late Cool). The different letters indicate significant differences between treatments.

Root type	Treatment	Initiation	Cessation	Max *l*_area_	Max Δ*l*_area_
Short	Cool	42 ± 5	112 ± 8	87 ± 12	52 ± 5
	Warm	57 ± 13	120 ± 0	78 ± 20	77 ± 10
	ECLW	42 ± 13	91 ± 15	67 ± 17	42 ± 13
	EWLC	42 ± 10	108 ± 8	63 ± 17	51 ± 19
Long	Cool	37 ± 6	120 ± 0b	90 ± 16	67 ± 8
	Warm	55 ± 22	120 ± 0b	75 ± 24	81 ± 13
	ECLW	47 ± 8	105 ± 7a	62 ± 5	62 ± 5
	EWLC	32 ± 5	120 ± 0b	77 ± 12	47 ± 8

During GS2 (and D2 following it), the largest but non-significant difference in short root cessation was between ECLW and Warm, which ceased four weeks later than ECLW (*P*_T_ = 0.213, *P*_adj_ = 0.284) (note: D2 started L105 days after the start of GS2) (Table 2). During GS2 and D2, long root growth ceased earliest in ECLW (*P*_T_ = 0.015, *P*_adj_ = 0.043) (Table 2). The root growth of every seedling slowed down in D2. However, at the end of GS2, short root cessation was not observed in two seedlings in Cool and Warm, and in one seedling in EWLC. In addition, long root cessation was not observed in two seedlings in Warm and EWLC, and in one seedling in Cool and ECLW, but for the statistical testing, the root growths were marked to end at the last imaging interval.

The time of maximum *l*_area_ of short roots (*P*_T_ = 0.755) and long roots (*P*_T_ = 0.695) did not differ significantly between treatments in GS2 (Table 2). The time of maximum Δ*l*_area_ of short roots (*P*_T_ = 0.281) and long roots (*P*_T_ = 0.130) did not show treatment differences either (Table 2).

### 3.2. Root growth and mortality

**Figure 2.**
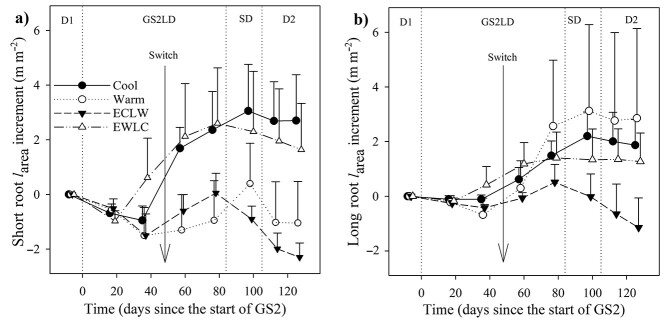
Increment of standing length (*l*_area_, length per unit image area) of (a) short roots and (b) long roots of silver birch seedlings in the experiment with similar air and soil conditions during the first growing season and dormancy periods (D1 and D2), and different soil temperatures during the second growing season (GS2), with long-day (LD) and short-day (SD) phases (Cool = constant 10 °C, Warm = constant 18 °C, ECLW = Early Cool & Late Warm, EWLC = early Warm & late Cool). The arrow indicates the time of the temperature switch in ECLW and EWLC. Bars indicate standard errors (*n* = 4).

**Figure 3.**
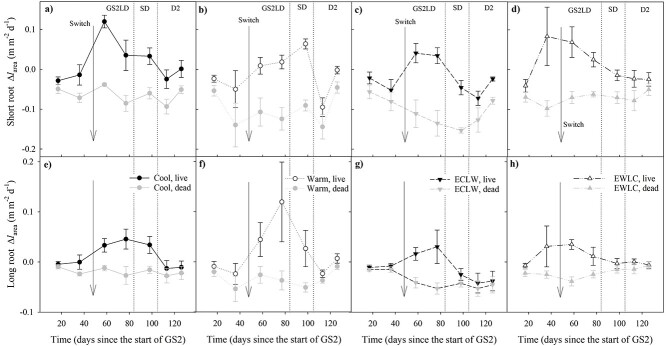
Net root-elongation rate (black lines) and mortality rate (gray lines) per unit image area (Δ*l*_area_) of (a–d) short roots and (e–h) long roots of silver birch seedlings in the experiment with similar air and soil conditions during the first growing season and dormancy periods (D1 and D2), and different soil temperatures during the second growing season (GS2), with long-day (LD) and short-day (SD) phases (Cool = constant 10 °C, Warm = constant 18 °C, ECLW = Early Cool & Late Warm, EWLC = Early Warm & Late Cool). The arrow indicates the time of the temperature switch in ECLW and EWLC. Bars indicate standard errors (*n* = 4). The first symbols show Δ*l*_area_ between −7 and 17 days from the start of GS2.

**Figure 4.**
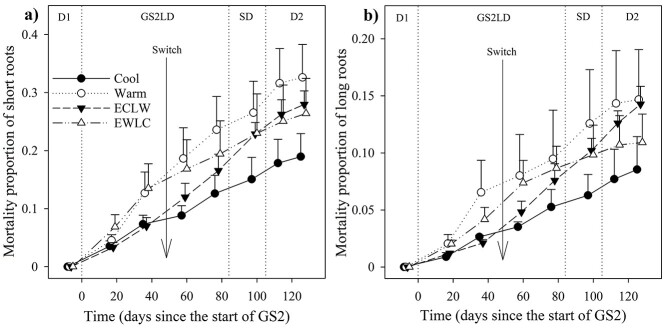
Proportion of dead root length of the sum of live root length and dead root length for (a) short roots and (b) long roots of silver birch seedlings after the first measurements of the growing seasons in the experiment with different soil temperatures during the second growing season (GS2), and with long-day (LD) and short-day (SD) phases (Cool = constant 10 °C, Warm = constant 18 °C, ECLW = Early Cool & Late Warm, EWLC = Early Warm & Late Cool). The arrow indicates the time of the temperature switch in ECLW and EWLC. Bars indicate standard errors (*n* = 4).

During GS2, the *l*_area_ of short roots increased more with time in Cool and EWLC than in ECLW, and more in Cool than in Warm (*P*_T × t_ = 0.039, estimates of fixed effects *P* ≤ 0.046) (Figure 2a). The long root *l*_area_ increment decreased more in ECLW than in Warm (*P*_T × t_ = 0.037, estimates of fixed effects *P* = 0.005), but Cool and EWLC remained intermediate (Figure 2b). The effect of soil temperature switch was clearest in ECLW in both short and long root *l*_area_ (Figure 2), and it was related to increasing root mortality (Figures 3 and 4).

Root production patterns after the start of GS2 were usually unimodal, the fastest short root growth taking place around the middle of the GS2, slightly later in Warm than in the other treatments (Figure 3). When mortality exceeded growth, it led to a decreasing *l*_area_ (Figures 2 and 3). The less negative trend in root production during D2 coincided with the decreasing root mortality (Figures 3 and 4).

During GS2, the mortality share of short roots increased less in Cool than in Warm and ECLW, and less in EWLC than in Warm (*P*_T × t_ = 0.024, estimates of fixed effects *P* ≤ 0.045) (Figure 4a). The effect of the temperature switch was clear, because EWLC and ECLW showed an opposite trend in root mortality share: for EWLC, a faster increase of short root mortality share in early GS2, and a slower increase at the end of GS2 and during D2—and the opposite in ECLW, but ending with a somewhat similar share at the end of the experiment. During GS2, the mortality share of long roots increased least in Cool, which differed from ECLW (*P*_T × t_ = 0.152, estimates of fixed effects *P* = 0.042) and slightly from Warm (estimates of fixed effects *P* = 0.100) (Figure 4b). The mortality shares of long roots in ECLW and EWLC had similar non-significant trends with soil temperature switch to those in short roots. The share increased quickly in EWLC during early GS2, and the increase slowed down towards the end of GS2 and in D2. However, the opposite trend occurred in ECLW (Figure 4b).

### 3.3. Survival analysis of roots

**Figure 5.**
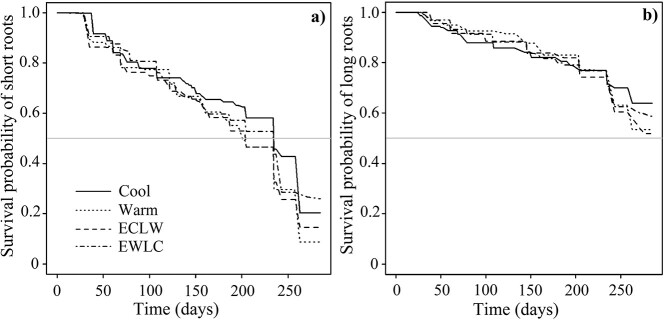
Survival curves of (a) short roots and (b) long roots of silver birch seedlings in the experiment with two growing seasons and two dormancy periods, with different soil temperatures during the second growing season (GS2) (Cool = constant 10 °C, Warm = constant 18 °C, ECLW = Early Cool & Late Warm, EWLC = Early Warm & Late Cool). Each curve is based on four seedlings. Median root longevity is obtained at a survival probability of 0.5, indicated by the gray horizontal line (cf. Table 3).

Survival distributions were not equal in the treatments based on short root data covering the entire study period from the start of GS1 to the last root imaging session in D2. Short roots in Cool and EWLC died later than randomly expected by the statistical test, and those in ECLW and in Warm earlier than randomly expected, the largest difference being between Cool and ECLW (*P* < 0.001) (Figure 5a). In the cohort including only short roots that appeared before the start of GS2, there was a similar significant effect (*P* < 0.001). This was also the case for the cohort including only the short roots that appeared during or after GS2 (*P* < 0.001), as well as for the cohort including short roots that appeared after the time of the soil temperature switch (*P* < 0.001). The corresponding full data of long roots did not show significant differences in survival probability between treatments (*P* = 0.477) (Figure 5b), nor did the data of the cohorts (*P* = 0.457, *P* = 0.592 and *P* = 0.906 for long roots appearing before and after the start of GS2, and after the time of the soil temperature switch, respectively). Based on the data covering the whole study period, the longevity of short roots was 7–8 months and the median longevities were one month longer in Cool and EWLC than in Warm and ECLW (Table 3). The mean long root longevity was nearly eight months, and it did not differ significantly between treatments (Table 3).

**Table 3:** Median and mean short and long root longevities (days with 95% confidence intervals) of silver birch seedlings in the experiment with two growing seasons (GS1, GS2) and a dormancy period, with different soil temperatures during GS2 (Cool = constant 10  °C, Warm = constant 18  °C, ECLW = Early Cool & Late Warm, EWLC = Early Warm & Late Cool). The different letters indicate significant differences among the treatments (i.e., no overlap in confidence interval). Median values could not be defined for long roots (cf. Figure 5).

	Short root		Long root
Treatment	Median	Mean	Mean
Cool	235 ± 3b	196 ± 5c	238 ± 7
Warm	200 ± 6a	180 ± 5ab	241 ± 8
ECLW	204 ± 4a	179 ± 4a	237 ± 7
EWLC	234 ± 5b	190 ± 4bc	239 ± 6

The fine root turnover rates of short roots based on the inverse of median longevity estimates were 1.6, 1.8, 1.8 and 1.6 year^−1^, and based on mean longevity estimates, 1.9, 2.0, 2.0 and 1.9 year^−1^ in Cool, Warm, ECLW and EWLC, respectively. For long roots, the root turnover rates based on mean longevity estimates were 1.5 year^−1^ in all treatments.

### 3.4. Shoot phenology

Soil temperature treatments did not significantly affect shoot phenology. The initiation of diameter growth occurred on average 29.8 ± 0.8 days after the start of GS2 (*P*_T_ = 0.517). In GS2, the maximum diameter growth rate was reached in GS2 on day 54.7 ± 3.9 (*P*_T_ = 0.366), and diameter growth ceased on day 97.4 ± 2.0 (*P*_T_ = 0.885).

The budburst of the top apical bud (=the initiation of shoot height growth) occurred 18.8 ± 1.0 days after the start of GS2 (*P*_T_ = 0.367). In GS2, the maximum height growth rate appeared on day 52.8 ± 3.7 (*P*_T_ = 0.104), and height growth ceased in GS2 on day 93.1 ± 1.5 (*P*_T_ = 0.248).

### 3.5. Shoot growth

**Figure 6.**
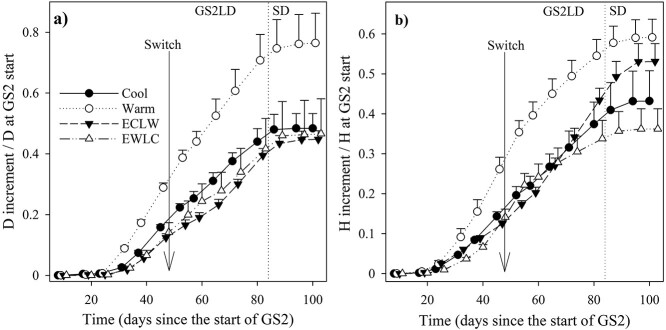
Diameter (a) and height (b) increments of silver birch seedlings in proportion to their sizes at the start of the treatment growing season, GS2. The experiment had different soil temperatures during the second growing season (GS2), with long-day (LD) and short-day (SD) phases (Cool = constant 10 °C, Warm = constant 18 °C, ECLW = Early Cool & Late Warm, EWLC = Early Warm & Late Cool). The arrow indicates the time of the temperature switch in ECLW and EWLC. Bars indicate standard errors (*n* = 4).

In GS2, the proportional diameter increment was greater in Warm than in ECLW, EWLC and Cool on days L59, L66 and L74, respectively (*P*_T_ = 0.050, *P*_t_ < 0.001, *P*_T × t_ < 0.001, *P*_adj_ ≤ 0.039) (Figure 6a). Before the soil temperature switch and until day E47, proportional shoot elongation increased more in Warm than in the other treatments (*P*_T_ = 0.339, *P*_t_ < 0.001, *P*_T × t_ < 0.001, estimates of fixed effects *P* < 0.001) (Figure 6b). During days L52–94, the proportional shoot elongation slowed down more in EWLC than in the other treatments (*P*_T_, *P*_t_ and *P*_T × t_ < 0.001, estimates of fixed effects *P* ≤ 0.011) and increased more in ECLW than in the other treatments (estimates of fixed effects *P* < 0.001). In pairwise comparisons, the height increment was greater in Warm than in the other treatments during days L59–74, but after this, Warm and ECLW no longer differed, and on day L94, the increment in ECLW was greater than in EWLC (*P*_adj_ ≤ 0.050) (Figure 6b). After the soil temperature switch, the proportional height increased more than the proportional diameter in ECLW (Figure 6). At the start of GS2, the heights of the seedlings were 91 ± 2.4, 102 ± 3.9, 102 ± 9.6 and 94 ± 1.4 cm in Cool, Warm, ECLW and EWLC, respectively. The diameters were 7.8 ± 0.37, 8.2 ± 0.33, 8.3 ± 0.72 and 8.0 ± 0.84 mm, respectively.

### 3.6. Biomass allocation

**Figure 7.**
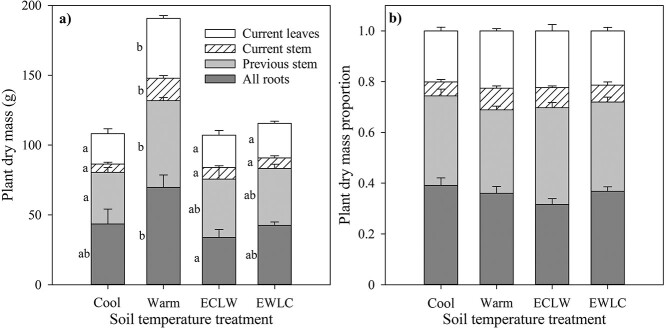
Dry mass (a) and its proportions (b) of silver birch seedlings among plant parts in soil temperature treatments (*n* = 4). All leaves of the treatment growing season (GS2) were combined, the stem with branches grown during GS2 (current) was separated from the previous growth, and the roots included all roots present after GS2 (upscaled from sector samples + roots separated from the rest of the containers). The different letters by cohorts indicate significant differences between soil temperature treatments (Cool = constant 10 °C, Warm = constant 18 °C, ECLW = Early Cool & Late Warm, EWLC = Early Warm & Late Cool).

At final harvest, the total dry mass of the birch seedlings was ca. 80% higher in Warm than in the other treatments (*P*_T_ = 0.003, *P*_adj_ ≤ 0.015), which did not differ from each other (Figure 7a). Root dry mass in Warm was double that of ECLW (*P*_T_ = 0.032, *P*_adj_ = 0.037) and non-significantly 50% greater than in Cool and EWLC (*P*_adj_ ≥ 0.161) (Figure 7a). The dry mass of stem parts and leaves was 50–170% greater in Warm than in the other treatments (*P*_T_ ≤ 0.014, *P*_adj_ ≤ 0.020; for previous growing seasons’ stems only Warm vs Cool was significant). The allocation of plant dry matter to leaves, stems and roots did not differ between the soil temperature treatments (*P*_T_ ≥ 0.194). Nor did the proportion of current stem to total stem dry mass (*P*_T_ = 0.299) (Figure 7b) differ. Root, stem and leaf biomasses accounted for 32–39, 41–46 and 20–23% of the total biomass, respectively (Figure 7b).

### 3.7. Linkages of root and shoot phenology

**Figure 8.**
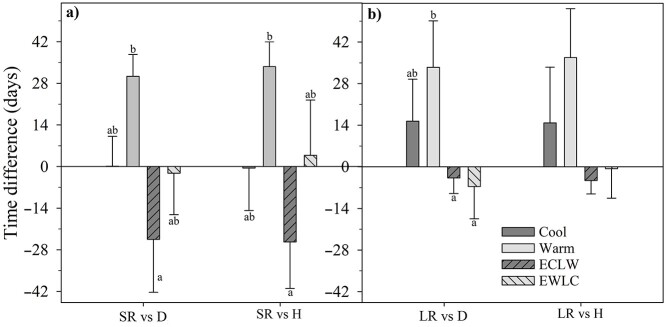
Time difference in maximum rate of root growth and shoot growth (stem diameter D and shoot length H) for (a) short roots (SR) and (b) long roots (LR) in the experiment with silver birch seedlings, with similar conditions during the first growing season (GS1) and with different soil temperatures during the second growing season (GS2) (Cool = constant 10 °C, Warm = constant 18 °C, ECLW = Early Cool & Late Warm, EWLC = Early Warm & Late Cool). A positive value indicates that root growth peaked later than shoot growth. The different letters indicate significant differences between the treatments within the growing seasons. Bars indicate standard errors (*n* = 4).

The offset values were calculated based on the timing of maximum root and shoot growth rates (reported above). During GS2, the offset values showed some treatment differences. Short root growth peaked later than stem diameter growth in Warm and earlier in ECLW (*P*_T × GS_ = 0.049, *P*_adj_ = 0.002) (Figure 8a). There was also a similar significant trend in the time difference between the peaks of short root and shoot elongation rates (*P*_T × GS_ = 0.043, *P*_adj_ = 0.012) (Figure 8a).

In GS2, long-root growth peaked later than stem diameter growth in Warm, but slightly earlier in ECLW and EWLC (*P*_T × GS_ = 0.081, *P*_adj_ ≤ 0.040 for the differences Warm vs ECLW and EWLC) (Figure 8b). Long root growth peaked later than shoot height growth in Warm. It was slightly earlier in ECLW during GS2 (*P*_T × GS_ = 0.043, *P*_adj_ = 0.117) (Figure 8b). The peaks of both short and long root growth compared with those of shoot elongation were observed later in GS2 than in GS1 in Warm (−8 days in GS1 vs 34–37 days in GS2, *P*_adj_ ≤ 0.023).

### 3.8. Root morphology and hydraulic conductivity

The largest differences in most root traits appeared between Warm and ECLW, viz. total root length (*P*_T_ = 0.050, *P*_adj_ = 0.052), length of roots with *d* ≤ 0.5 mm (*P*_T_ = 0.041, P_adj_ = 0.042), length of roots with 0.5 < *d* ≤ 1 mm (*P*_T_ = 0.056, *P*_adj_ = 0.055), and total root surface area (*P*_T_ = 0.080, *P*_adj_ = 0.087) were larger in Warm than in ECLW. The other root traits, including hydraulic conductivity, did not differ significantly between treatments (*P*_T_ ≥ 0.111) (Table 4).

**Table 4:** Root morphological traits and hydraulic conductance (mean ± SE) of silver birch seedlings after different soil temperature treatments at the end of the experiment. Root length in total and in root diameter (d) classes, surface area, volume and tip number were obtained by upscaling the sector sample data for the whole root system. Reverse-flow root hydraulic conductance was divided by root surface area for the conductivity. The different letters indicate significant differences between soil temperature treatments (Cool = constant 10 °C, Warm = constant 18 °C, ECLW = Early Cool & Late Warm, EWLC = Early Warm & Late Cool) (*df* = 3, *n* = 4, except 3 in SRL 2 < *d* < 4.5 mm in ECLW and EWLC).

Root trait	Cool	Warm	ECLW	EWLC
Total length, m	429 ± 73	651 ± 78	324 ± 86	507 ± 57
Length (d ≤ 0.5 mm), m	265 ± 43ab	405 ± 36b	198 ± 59a	319 ± 39ab
Length (0.5 < d ≤ 1 mm), m	129 ± 23	196 ± 29	95 ± 22	149 ± 17
Length (1 < d ≤ 2 mm), m	31 ± 7.2	44 ± 13	28 ± 5.3	35 ± 3.7
Length (2 < d ≤ 4.5 mm), m	3.5 ± 1.1	5.9 ± 2.2	2.5 ± 0.7	3.3 ± 0.8
Surface area, m^2^	0.77 ± 0.14	1.17 ± 0.18	0.59 ± 0.14	0.90 ± 0.09
Volume, cm^3^	114 ± 23	171 ± 33	90 ± 19	131 ± 13
Tips ×1000	84.2 ± 15.4	131 ± 13.4	76.6 ± 22.6	105 ± 11.9
Tips/length, cm^−1^	1.95 ± 0.04	2.04 ± 0.10	2.29 ± 0.14	2.08 ± 0.04
SRL (*d* ≤ 2 mm), m g^−1^	35.5 ± 3.0	41.4 ± 8.7	30.8 ± 3.5	36.7 ± 1.6
Tissue density (*d* ≤ 2 mm), kg m^−3^	95 ± 5.5	102 ± 6.7	100 ± 7.7	99 ± 3.9
Conductance, mg s^−1^ MPa^−1^	58.3 ± 19.1	116 ± 33.3	65.7 ± 15.9	86.1 ± 6.1
Conductivity, mg s^−1^ MPa^−1^ cm^−2^	74.9 ± 17.6	98.6 ± 27.1	119.5 ± 18.1	98.6 ± 12.3

### 3.9. EIS

**Figure 9.**
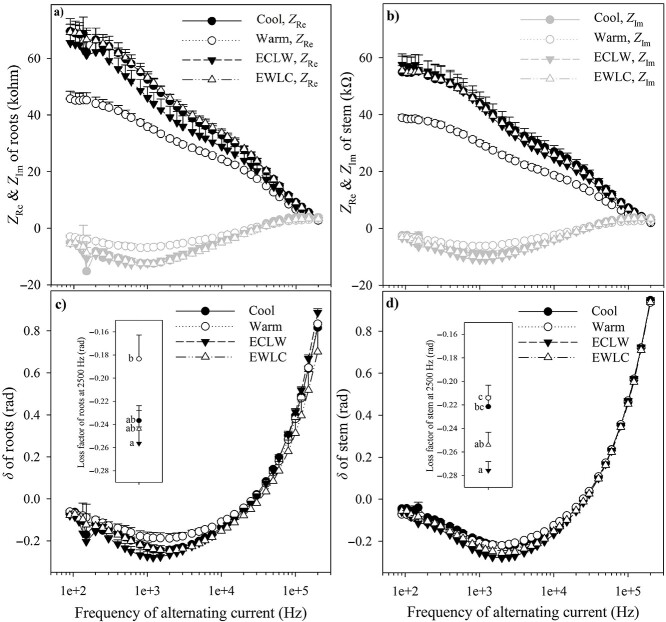
The mean real (*Z*_Re_) and imaginary (*Z*_Im_) parts of impedance of silver birch roots (a) and stems (b), and the impedance loss factors (*δ*) of roots (c) and stem (d). Bars show SE (*n* = 8). Panels (a) and (b) share the legends, as well as (c) and (d). Soil temperature treatments were Cool = constant 10 °C, Warm = constant 18 °C, ECLW = Early Cool & Late Warm and EWLC = Early Warm & Late Cool. The different letters indicate significant treatment differences in the *δ* at 2.5 kHz, shown in the inserts of the panels (c) and (d) (*P* < 0.05).

The real parts of electrical impedance of both roots and stem were lower in Warm than in the other treatments (Figure 9a–b), but the differences were less clear in the imaginary parts (Figure 9a–b). Loss factors changed from negative to positive; i.e., impedance changed from capacitive to inductive, with frequencies higher than 30 kHz, except in EWLC roots higher than 40 kHz and ECLW stem higher than 25 kHz (Figure 9c–d). At a frequency of 2.5 kHz, the loss factors of roots and shoots were highest in Warm. The largest differences were found in roots compared with ECLW (*P*_T_ = 0.023, *P*_adj_ = 0.027) (Figure 9c) and in stem to ECLW and EWLC (*P*_T_ < 0.001, *P*_adj_ ≤ 0.041) (Figure 9d). The loss factor of the stem was greater in Cool than in ECLW (*P*_adj_ = 0.003) (Figure 9d). The loss factor at 2.5 kHz was slightly lower in the roots than stem in Cool, but slightly higher in the roots than stem in the other treatments, and the difference between roots and stem was largest in Warm (*P* ≥ 0.100) (Figure 9c–d). In the CLAFIC analysis, all the real and imaginary parts of impedance and the phase angle of impedance in roots and stem were classified according to the treatments with 3 ≤ *k* ≤ 6 (Figure S2 available as Supplementary data at *Tree Physiology* Online).

### 3.10. Foliar nutrients and soil characteristics

The C content of short-shoot leaves was greatest in ECLW (*P*_T_ = 0.002, P_adj_ ≤ 0.029) at both sampling times in GS2, and it increased between them, viz. after the soil temperature switch (*P*_adj_ = 0.046) (Table 5). In long-shoot leaves sampled at L81 days after the start of GS2, C content was higher in ECLW than in Cool (*P*_T_ = 0.024, *P*_adj_ = 0.031).

**Table 5:** Mean (±SE, *n* = 4) C and N contents (mg g^−1^) and their ratio in short-shoot (SS) leaves of silver birch seedlings sampled 44 and 78 days after the start of the second growing season (GS2), and in long-shoot (LS) leaves in soil temperature treatments (Cool = constant 10  °C, Warm = constant 18  °C, ECLW = Early Cool & Late Warm, EWLC = Early Warm & Late Cool) sampled 81 days after the start of GS2 (near the end of the long-day phase). The different lowercase letters indicate significant differences between treatments; the different uppercase letters indicate significant differences between the sampling times in SS leaves within the treatments.

	Leaf/Day	Cool	Warm	ECLW	EWLC
C	SS/44	494 ± 6.0a	487 ± 2.4a	502 ± 3.9bA	493 ± 6.5a
	SS/78	490 ± 3.3a	485 ± 4.3a	516 ± 1.5bB	483 ± 7.2a
	LS/81	476 ± 2.5a	479 ± 2.0ab	492 ± 3.0b	485 ± 5.0ab
N	SS/44	15.9 ± 1.06a	23.0 ± 1.77bB	15.1 ± 0.84a	21.5 ± 3.33abB
	SS/78	12.9 ± 1.82	11.6 ± 0.73A	15.6 ± 0.62	11.8 ± 1.11A
	LS/81	12.3 ± 1.96a	12.3 ± 0.75a	18.8 ± 1.40b	12.4 ± 1.44ab
C/N	SS/44	31.5 ± 2.0abA	21.5 ± 1.7aA	33.6 ± 1.6b	24.7 ± 4.0abA
	SS/78	40.0 ± 4.8B	42.1 ± 2.4B	33.2 ± 1.3	42.0 ± 3.5B
	LS/81	41.0 ± 5.0	39.2 ± 2.2	26.6 ± 2.0	40.5 ± 3.8

The N content of short-shoot leaves was greater in Warm than in Cool and ECLW on day E44 (*P*_adj_ ≤ 0.032). The N content of short-shoot leaves decreased between days E44 and L78 in EWLC and Warm (*P*_t_ < 0.001, *P*_T × t_ = 0.004, *P*_adj_ < 0.001) (Table 5). The C/N ratio of short-shoot leaves was higher on day L78 but remained the same in ECLW (*P*_t_ < 0.001, *P*_T × t_ = 0.001). The short-shoot leaf C/N ratio was higher in Warm than in ECLW on day E44 (*P*_adj_ = 0.047). The N content of long-shoot leaves was greatest in ECLW (*P*_T_ = 0.018, *P*_adj_ = 0.050 for ECLW vs Cool and Warm, *P*_adj_ = 0.052 for ECLW vs EWLC), while the C/N ratio was lowest (*P*_T_ = 0.037, *P*_adj_ ≤ 0.148).

Among the other nutrients (B, Ca, Cu, Fe, K, Mg, Mn, P, S, Zn) of long-shoot leaves sampled near the end of the long-day phase, K and Cu content was greater in ECLW than in EWLC (*P*_T_ ≤ 0.033, *P*_adj_ ≤ 0.041), Fe content was greatest in ECLW (*P*_T_ < 0.001, *P*_adj_ ≤ 0.011) (Table S2 available as Supplementary data at *Tree Physiology* Online).

The foliar C, N, P, Mg and Mn amounts per seedling were greatest in Warm (*P*_T_ ≤ 0.005, *P*_adj_ ≤ 0.034), K, Ca and Cu amounts were greater in Warm than in Cool and EWLC (*P*_T_ ≤ 0.007, *P*_adj_ ≤ 0.016), and Zn and B amounts were greater in Warm than in EWLC (*P*_T_ = 0.022, *P*_adj_ = 0.045) (Table S3 available as Supplementary data at *Tree Physiology* Online).

At the end of the experiment, loss on ignition (LOI) was on average 11.1 ± 0.33% in the soil organic layer and 2.4 ± 0.09% in mineral soil. It did not differ significantly between soil temperature treatments (*P*_T_ = 0.167). In mineral soil, LOI was highest in the uppermost 0–10 cm layer, intermediate in the 20–30 cm layer, and lowest in the 10–20 cm layer (*P* < 0.001, *P*_adj_ ≤ 0.015) (Table S4 available as Supplementary data at *Tree Physiology* Online).

At the end of the experiment, the soil pH did not differ significantly between treatments (*P*_T_ = 0.250). The soil organic layer had a lower pH than mineral soil at a depth of 10–30, and the pH was lower in a mineral soil layer of 0–10 cm than 20–30 cm (*P* < 0.001, *P*_adj_ ≤ 0.026) (Table S4 available as Supplementary data at *Tree Physiology* Online). These differences in SOM and pH were significant but quite small.

### 3.11. Soil gases

**Figure 10.**
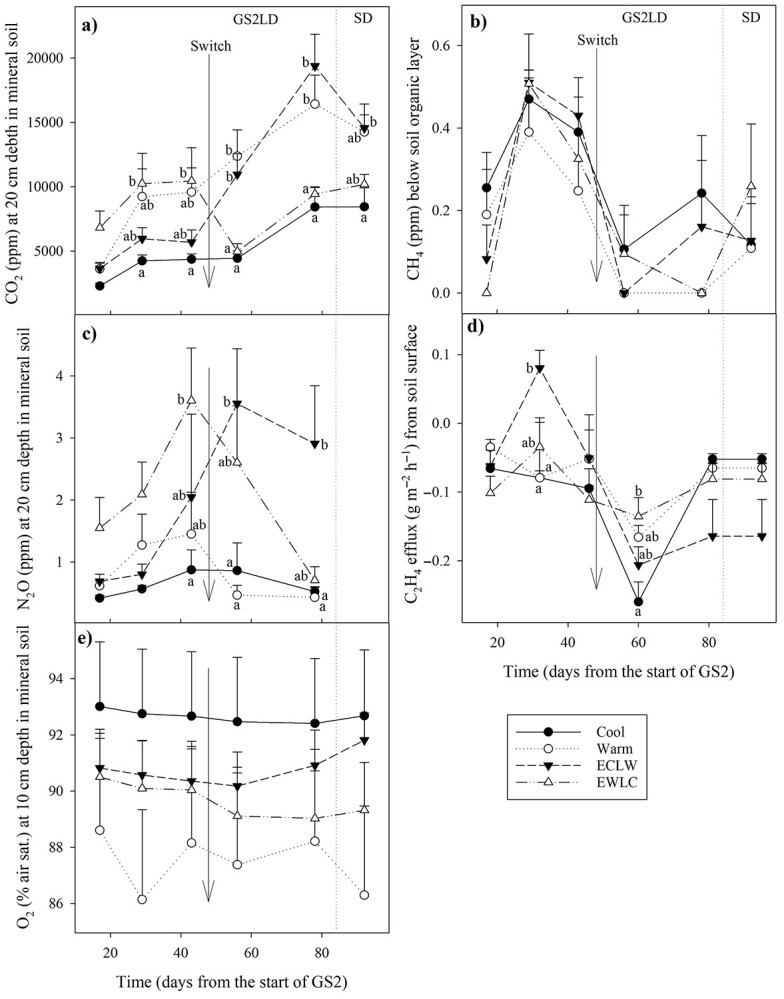
Gas concentrations and fluxes in the soil of the silver birch experiment, which had similar air and soil conditions during the first growing season and different soil temperatures during the second growing season (GS2), with long-day (LD) and short-day (SD) phases (Cool = constant 10 °C, Warm = constant 18 °C, ECLW = Early Cool & Late Warm, EWLC = Early Warm & Late Cool). The arrow indicates the time of the temperature switch in ECLW and EWLC. Bars indicate standard errors (*n* = 4). The different letters indicate significant differences between treatments (*P* < 0.05).

There was a tendency for CO_2_ concentrations to increase with the soil temperature, and the effect of the soil temperature switch was clear (Figure 10a). The carbon dioxide concentration in the mineral soil was higher in EWLC than in Cool on days E30 and E44 after the start of GS2, but after the soil temperature switch, the concentration was greater in Warm and ECLW than in Cool and EWLC. In the last measurement, the difference was significant only between Cool and ECLW (*P*_T × t_ < 0.001, *P*_adj_ ≤ 0.043) (Figure 10a). The CO_2_ concentration below the soil organic layer showed a similar trend, but with smaller differences (*P*_T × t_ < 0.001). The largest difference was between EWLC and ECLW on E44 days after the start of GS2 *P*_adj_ = 0.052 (result not shown). The pairwise differences in CO_2_ concentrations before and after the soil temperature switch (day E44 vs L57) were significant in the mineral soil within both ECLW and EWLC (Figure 10a), and below the soil organic layer within EWLC (*P*_adj_ < 0.001).

In the mineral soil, the CH_4_ concentration was 0.09–0.15 ppm in Warm, but CH_4_ was not detected in the other treatments (no statistical testing). The CH_4_ concentration on top of the mineral soil below the soil organic layer did not differ between treatments (*P*_T × t_ = 0.347) (Figure 10b).

The N_2_O concentration in the mineral soil remained quite stable in Cool and Warm, and was at its greatest in ECLW. It decreased in EWLC after the soil temperature switch (*P*_T × t_ = 0.035, *P*_adj_ ≤ 0.030) (Figure 10c). There were no consistent changes with soil temperature treatments in the N_2_O concentration below the soil organic layer, although the EWLC pattern differed from the others for some reason (*P*_T × t_ < 0.001, *P*_adj_ ≤ 0.018) (result not shown).

The soil acted as a C_2_H_4_ sink except on day E32 in ECLW, which differed from Cool and Warm, and the sink was weaker in EWLC than in Cool on day L60 (*P*_T × t_ = 0.003, *P*_adj_ ≤ 0.035) (Figure 10d). The C_2_H_4_ sink was at its strongest in Cool on day L60 (*P*_adj_ ≤ 0.028), in Warm, it was stronger on day L60 than on day E18 (*P*_adj_ = 0.043), and in ECLW, it was stronger on day L60 than earlier and weakest on day E32 (*P*_adj_ = 0.050), with the exception of day E46, which did not differ significantly. In Warm, days E18 and L60 differed (*P*_adj_ = 0.043). In EWLC, there was no significant difference with time.

Oxygen concentration in mineral soil fluctuated from 86 to 93% of air saturation and did not differ between treatments (*P*_T × t_ < 0.001, *P*_adj_ ≥ 0.362), but the variation between days was lowest in Cool and highest in Warm (Figure 10e).

### 3.12. Results during the acclimation growing season

No significant differences in the root phenology occurred between the ‘treatments’ (not yet actualized) in the GS1 acclimation period (*P*_adj_ ≥ 0.066) (Table S5 available as Supplementary data at *Tree Physiology* Online). During GS1, the *l*_area_ increment of short roots (*P*_*“*T” × t_ = 0.794) or long roots (*P*_*“*T” × t_ = 0.552) with time did not differ significantly between the ‘treatments’ (results not shown). The mortality share of short roots did not differ either (*P*_*“*T” × t_ = 0.587) (result not shown), but the mortality share of long roots increased more in ‘Cool’ than in ‘Warm’ and ‘ECLW’ towards the end of GS1 (*P*_*“*T” × t_ = 0.110, estimates of fixed effects *P* ≤ 0.049) in D1, i.e., 7 days before GS2 started, 0.10 ± 0.015, 0.037 ± 0.015, 0.07 ± 0.014 and 0.07 ± 0.028 in Cool, Warm, ECLW and EWLC, respectively.

The initiation of diameter growth occurred on average 28.8 ± 0.7 days after the start of GS1 (similar conditions) (*P*_*“*T”_ = 0.248). The maximum diameter growth rate was reached in GS1 on day 34.9 ± 0.4 (*P*_*“*T”_ = 0.426), and diameter growth ceased on day 92.7 ± 1.7 (*P*_*“*T”_ = 0.710). The budburst of the top apical bud (=the initiation of shoot height growth) occurred 22.7 ± 0.5 days after the start of GS1 (*P*_*“*T”_ = 0.627). In GS1, the maximum height growth rate appeared on day 42.4 ± 4.5 (*P*_*“*T”_ = 0.222). Shoot height growth ceased in GS1 on day 83.6 ± 3.2 (*P*_*“*T”_ = 0.782).

The offset values (difference of the timing of maximum root and shoot growth rates) did not show ‘treatment’ dependency during the acclimation GS1 (*P*_adj_ = 1.000). In GS1, short root growth peaked on average 13.6 ± 0.4 days later than diameter growth and 6.1 ± 4.5 days later than shoot elongation. The respective values for long root growth were 10.9 ± 1.8 and 3.4 ± 4.7 days. The peaks of short root growth and stem diameter growth differed between growing seasons in ECLW (14 days in GS1 vs −25 days in GS2, *P*_T × GS_ = 0.049, *P*_adj_ = 0.007). The peaks of both short and long root growth compared with those of shoot elongation were observed later in GS2 than in GS1 in Warm (−8 days in GS1 vs 34–37 days in GS2, *P*_T × GS_ ≤ 0.076, *P*_adj_ ≤ 0.023).

## Discussion

### 4.1. Root and shoot growth dynamics

In field conditions, soil temperature follows air temperature with a lag. In boreal forests, the aboveground tree parts grow earlier and the roots mostly later in the growing season when soil remains warm. Our novel idea was to test whether this is driven by resource competition between shoots and roots or whether soil warming during the growing season increases the root growth. We aimed to break the linkage of air and soil temperature and test the effects of soil temperature in the control of root and shoot growth dynamics. This was achieved by switching soil temperature crosswise between low and high in two treatments in the middle of the growing season (Early Cool + Late warm, ECLW and Early Warm + Late Cool, EWLC), the other treatments being constantly Cool (10 °C) and Warm (18 °C) soil. Air conditions were similar in all the treatments. The effect of the soil temperature switch was prominent in short root mortality, which increased with soil warming (ECLW) and decreased with soil cooling (EWLC). Short root mortality was highest in Warm and lowest in Cool, which was in accordance with the results observed in fine roots (Leppälammi-Kujansuu et al. 2014, Kilpeläinen et al. 2019). Long root mortality had the same patterns, but the changes were smaller and not significant. As observed also in previous studies, short roots are evidently more dynamic and sensitive to environmental changes than long roots, thus explaining the differences in mortality (McCormack et al. 2015). The ectomycorrhizal symbiosis in the short roots may also be sensitive to higher temperatures, while they are more tolerant to low temperatures (Kilpeläinen et al. 2020). The high mortality may also relate to the limited growth space in the containers, but the lower average fine root biomass (ca. 64 g m^−2^) than in a mixed sapling stand in southern Finland (ca. 98 g m^−2^, Kalliokoski et al. 2010) did not support this.

The growth of both short and long roots was quite low compared with mortality in all the treatments, and increasing root growths often coincided with decreasing root mortalities. As the combination of growth and mortality, the standing length of short roots increased during the treatment growing season (GS2) most in Cool and EWLC, and least in ECLW and Warm. Net root growth was even greater at a soil temperature of 10 than 18 °C, because in warm soil, root mortality exceeded growth. It is known that a major part of tree root production is concentrated in the late growing season in the boreal zone (Abramoff and Finzi 2015, Ding et al. 2020). Our hypothesis that abundant root production driven by high soil temperature would be concentrated in the late growing season in ECLW and in the early growing season in EWLC did not actualize in silver birch with the applied soil temperatures. Here, soil temperature treatments had no large effects on the production patterns of short and long roots, which unimodally peaked around the mid growing season.

The initiation of short and long root growth was not affected by the soil temperature treatments. This can be explained by simultaneous soil warming in all treatments above the threshold for root growth (≈ 6 °C, according to Alvarez-Uria and Körner (2007)), which is lower than the lowest treatment temperature (10 °C). In cool climates, the soil as a temperature buffer follows the air temperature with a delay and starts to warm later than the air in the spring, which may delay root growth until the later spring or early summer (Steinaker and Wilson 2008). Correspondingly, the soil remains warm in relation to the air in the autumn and supports sustained root growth. However, in contrast with our expectations, long root growth ceased earliest in ECLW, which is probably connected with the increased root mortality after the soil temperature switch.

The results of root longevities matched the observed root mortality well. Short roots in Cool and EWLC died a month later than in ECLW and Warm measured as median longevities (Cool and EWLC: 234–235 days, Warm and ECLW: 200–204 days). Based on the median longevities, the short root turnover was higher in Warm and ECLW (1.8 year^−1^) than in Cool and EWLC (1.6 year^−1^). In field studies, in a mature silver birch stand in northern Finland, the fine root turnover rate 1.06 year^−1^ (Ding et al. 2019) was much lower than in our study. The difference is a consequence of the laboratory conditions, where the annual cycle was run faster than it would proceed in field conditions with long winters in the boreal zone. However, the laboratory results therefore reveal the proportional differences between treatments. Root turnover varies considerably with tree species and growing conditions. Based on a compilation of 30 studies in boreal forests, it was on average 1.30 ± 0.35 year^−1^ (mean ± SE; calculated as annual production/mean annual biomass) (Finér et al. 2011).

The soil temperature treatments were also prominently reflected in the aboveground growth of silver birch seedlings. The timing of budburst did not differ between the treatments, but the following aboveground growth of the seedlings was significantly affected. The soil temperature switches significantly affected the shoot elongation rate, which accelerated in ECLW and slowed down in EWLC after the switch. The relative stem diameter and height of the seedling at the start of GS2 increased most in Warm. At the start of GS2, the seedlings in Warm were slightly larger than in EWLC, which might explain their different development in similar conditions before the soil temperature switch. The difference lasted despite the accelerated shoot elongation in ECLW towards the end of the experiment. The proportional diameter growth was not affected by the soil temperature switch, and stem elongation was therefore more sensitive to the soil temperature change in the middle of the growing season than diameter growth. However, the timing of initiation, the maximum rate or the cessation of diameter and height growth did not differ significantly between treatments. Aphalo et al. (2006) also reported remarkable shoot growth suppression of silver birch in cool than warm soil. In a hydroponic experiment with root zone temperatures of 2 °C, 6 °C, 12 °C and 17 °C, the shoot and root growth of silver birch seedlings were greatest at the highest temperature (Solfjeld and Johnsen 2006).

When the timing of maximum root and shoot growths was compared, short root growth peaked later than stem diameter growth in Warm. However, in ECLW, short root growth peaked earlier than stem diameter growth and root production decreased most towards the end of GS2. Therefore, the hypothesis of strong control of soil temperature on root growth was not supported, but the result agrees with the view that due to competing sinks, root and shoot production may not peak simultaneously (Lyr and Hoffmann 1967, Iivonen et al. 2001). Based on a review of twenty studies, the fine root growth of boreal trees peaked on average 48 ± 8 days later than shoot growth (Abramoff and Finzi 2015). According to the same study, root growth peaked 44 ± 12 days earlier in deciduous tree species than in conifers, which may be due to the prolonged assimilation activity of needles vs senescing leaves in broadleaves. Our results thus support the asynchrony of root and shoot phenology (Lyr and Hoffmann 1967, Steinaker et al. 2010, Abramoff and Finzi 2015, Sloan et al. 2016, Radville et al. 2016*a*, Kilpeläinen et al. 2019). The root growth dynamics of silver birch seedlings were not only driven by soil temperature, but tree-intrinsic factors (resource competition among tree parts) also played a role.

At the end of the experiment, the seedlings in Warm clearly had the largest biomass, whereas the other treatments were close to each other. Although more short roots died in Warm than ECLW, Warm resulted in significantly larger root biomass compared to ECLW. The most likely reason for this is that Warm seedlings had more long roots than ECLW, contributing more to the biomass than short roots. Shoot dimensions at the beginning of GS2 were similar in Warm and ECLW, but cool soil in the early growing season resulted in smaller shoot growth in ECLW than in Warm. The smallest total biomass could have been expected to appear in Cool, with ECLW and EWLC intermediate, but no large differences were seen. This means that warm soil either in the first (EWLC) or the second half of the growing season (ECLW) did not boost silver birch growth sufficiently to result in tree biomasses differing significantly from Cool. This may be a result of the interaction of the leaves and roots. Leaves in ECLW and Cool grew poorly in the early summer, and their photosynthesis was low compared with EWLC and Warm (Kilpeläinen et al., in press), possibly leading to a low carbon allocation to the roots. The result may also be due to the soil temperature switch from cool to warm, which increased root respiration and was accompanied by the observed shortened root longevity in ECLW. Leppälammi-Kujansuu et al. (2014) observed that Norway spruce fine roots grown outside the active aboveground growing season had a shorter longevity than fine roots grown during the active growing season. In relation to their result, it can also be speculated that here the roots grown in cool soil were not sufficiently resilient and plastic to adjust to the abrupt soil warming, and therefore could not enhance biomass production.

Increased growth allocation to the roots in cool soil to enhance nutrient uptake was not supported, as growth allocation was not altered by the treatments. In this short-term experiment, the foliar concentrations of nitrogen and other nutrients did not indicate deficiency, which could explain the lack of allocation responses. However, based on several observational field studies with trees that have had a long time to acclimate and adapt to their growing conditions, significantly more growth is allocated to the roots in colder than warmer climates (Ostonen et al. 2007, Helmisaari et al. 2009, Ostonen et al. 2011, Leppälammi-Kujansuu et al. 2014, Finér et al. 2019). Direct nutrient responses can be clear, as was shown by a hydroponic study in which high N availability increased more in aboveground than belowground growth, and concurrently, low N led to a higher root/shoot ratio in Norway spruce seedlings (Kaakinen et al. 2004).

### 4.2. Root traits

One would have expected the roots to acclimate their morphology to enhance water and nutrient uptake in cool soil, where the availability is decreased. Here, the expectation was not supported, but the length of roots with a diameter of ≤ 0.5 mm and a total root surface area of ≤ 1 mm were slightly (*P* < 0.1) higher in Warm than in ECLW. Instead, the absorptive root biomass increased with decreasing temperature and nutrient availability e.g., in a gradient from temperate to boreal forests (Ostonen et al. 2017). In our study, the values of morphological root traits mostly decreased in the order Warm > EWLC > Cool > ECLW. Generally, high-specific root length (SRL), small root diameter and a large number of root tips per unit root length indicate a large absorbing surface area in relation to the construction cost of roots (Comas and Eissenstat 2004). The applied soil temperatures did not cause root damage that would show as clearly increased reverse flow hydraulic conductance (Korhonen et al. 2019). The slightly lowered reverse flow root hydraulic conductance implied low water transport capacity (Tyree et al. 1995) in the roots of Cool in contrast to Warm but may also be affected by root system size.

In the analysis of electrical impedance spectra (EIS), the lower real part of impedance (resistance) in the roots and stem in Warm implied a higher electrolyte content in sap in Warm than in the other treatments. In other words, increased electrolyte content enabled an easier passage of electric current. The difference in resistance between Warm and other treatments increased with decreasing frequency of alternating current. At low frequencies (<100 kHz), alternating current will not pass the lipid bilayer of cell membranes but will flow through apoplastic space, the resistance of which mostly contributes to the total impedance (Repo et al. 2012). The Warm treatment stood out also in the loss factor analysis. Previously, loss factor has been shown to change with freezing damage and apparent changes in cell electrolyte balance of Scots pine roots (Di et al. 2019). As the soil temperature treatments (10–18 °C) were insufficiently harsh to break the cell membranes in the roots and shoots, the electrolyte balance of the cells would not have changed dramatically. The loss factors of the roots and stems would therefore be in the same order, from smallest to largest in the treatments. This happened at a frequency of 2.5 kHz, where the loss factors of the roots and shoots were highest in Warm, and the largest differences were found in the roots compared with ECLW, and in the stem, with ECLW and EWLC. According to the CLAFIC analysis of the real and imaginary parts and the impedance phase angle of the roots and stem, the different treatments formed their own classes. The treatments therefore left their fingerprints on the electrolyte balance of cells, both in the roots and the stems. The treatment differences were clear but they could not be explained only by the mean value of an EIS set because the spectra were classified right when *k* > 1.

### 4.3. Foliar nutrients

The nutrient content of both short- and long-shoot leaves increased with soil temperature, which accords with previous studies showing enhanced nutrient availability and uptake to be reflected in increasing nutrient content in trees (Domisch et al. 2002). Before the soil temperature switch, the N content of short-shoot leaves was lowest in Cool and ECLW. After the switch, the N content of short-shoot leaves remained about the same in Cool and ECLW but was lower than in the first sampling in Warm and EWLC. The pattern of Warm and EWLC was similar to silver birches in middle Finland where N content in short-shoot leaves was lower on 10th June (ca. 36.5 mg g^−1^) than on 14th July (24.9 mg g^−1^) (Riikonen et al. 2003). However, the birches in a fertilized experimental field (Riikonen et al. 2003) sampled near to our days E44 and L78 had higher foliar N content than especially cool treatments (Cool and ECLW) in our study. The N content of long-shoot leaves was ca. 50% higher and many other nutrient contents (most prominently Fe) were also higher in ECLW than in the other treatments. This agrees with our expectations of better foliar nutrient condition in warm than cool soil, and the difference in short-shoot and long-shoot leaves resulting from the different timing of their emergence. The soil temperature switch from cool to warm evidently enhanced the nutrient uptake of ECLW seedlings, which was actualized as increased foliar nutrient content, enhanced water movement, stem elongation and leaf expansion rates (Kilpeläinen et al., in press). Presumably, seedlings in Warm would also have had a high nutrient content, but their nutrients were diluted in the double-sized foliage, and the nutrient amounts per tree were therefore highest in Warm. In a previous experiment with constant soil temperatures of 5 °C, 10 °C and 20 °C, foliar N content and amounts increased comparably from cool to warm, and N content was coherently lower in their 5 °C (Aphalo et al. 2006) than our 10 °C treatment.

### 4.4. Soil conditions

Soil organic matter and pH of the organic or mineral soil layers did not show significant treatment effects, although soil warming in the long run could have affected them, e.g., through altered root litter amounts and decomposition rates. There was a tendency for CO_2_ concentrations to be higher in warm than cool soil, as could have been expected. The soil temperature switches from 10 to 18 °C and the reverse were reflected in increasing and decreasing CO_2_ concentration in mineral soil, respectively. These differences in soil CO_2_ concentrations are probably connected with larger root respiration and microbial activity in warm than cool soil (Wei et al. 2010). Although fine root growth is usually enhanced at higher soil temperatures, it is simultaneously associated with earlier root senescence and shorter longevity, resulting from increased root respiration and from increased herbivore and pathogen activity (Eissenstat et al. 2000, McCormack and Guo 2014). Shorter root longevity means more root litter for decomposers. As soil warming increased root mortality, temperature acclimation of root respiration may not have happened on a large scale during the treatment growing season. The acclimation varies with biomes, tree species, root morphology and soil depths (Jarvi and Burton 2020, Noh et al. 2020) and seasonal acclimation (Burton and Pregitzer 2003) can be weaker than longer-term acclimation (Jarvi and Burton 2013).

Methane is mostly produced in organic soils and wet (anaerobic) conditions (Bridgham et al. 2013), whilst in the well-aerated mineral soil in our experiment, its concentration (and apparently also production) was low or not detected at all. High CO_2_ and CH_4_ concentrations in soil hinder root growth (Gilman et al. 1982, Gliński and Stępniewski 1985, Crawford 1989, Kim et al. 2017). The concentrations of CO_2_ (ca. 0.2–2%) and CH_4_ (ca. 0–0.5 ppm) in our soil were much lower than those at the same depth of 20 cm in the soils of Gilman et al. (1982) rich in CO_2_ (mean ca. 8%) and CH_4_ (ca. 3%). Moreover, as is typical of well-aerated mineral soils (Zechmeister-Boltenstern and Nikodim 1999), the soil acted mostly as an ethylene sink, with no distinctive differences between soil temperature treatments. Root growth is strongly inhibited by C_2_H_4_ (Arshad and Frankenberger 1992, Visser and Pierik 2007). However, all the measured gas concentrations of our experiment were probably too low to hamper roots.

Our results indicated soil temperature-dependent changes in N_2_O concentration in mineral soil, because it was highest in ECLW and decreased in EWLC after the soil temperature switch, which is in accordance with field studies indicating increasing N_2_O emissions with soil warming (Cui et al. 2018). Nitrous dioxide production is driven in soil mainly through nitrification and denitrification processes that can be affected by soil temperature, moisture and other soil conditions, and the composition of soil microbiota (Martins et al. 2017). Case-specific differences exist, and in our experiment, soil CO_2_ and N_2_O concentrations were higher than observed at the same depth, for example, in a 40-year-old Scots pine (*Pinus sylvestris* L.) forest in southern Finland (Pihlatie et al. 2007), but soil CO_2_ concentrations were comparable to the values observed by Pumpanen et al. (2003) in a Scots pine forest in southern Finland. Fender et al. (2013) detected a negative correlation between fine root surface area in rhizotrons and soil N_2_O emission. Conditions such as high soil CO_2_ concentration and soil frost leading to lower N uptake of plants can increase soil N_2_O emissions (Groffman et al. 2006, Kim et al. 2017, Repo et al. 2021) that further contribute to climate change.

## Conclusions

In conclusion, the root growth dynamics of silver birch seedlings were not only driven by the general trend for the soil to stay warm in relation to the air in the late growing season, but tree-intrinsic factors (resource competition among tree parts) also played a role. The soil temperature switches significantly affected shoot elongation. The study showed the importance of soil temperature in fine root dynamics not only through root growth but via root mortality. Soil warming has complex effects on tree and soil functioning, which can further affect carbon dynamics in forest ecosystems, with a feedback to climate change.

## Data and Materials Availability

Data and materials are available to academic researchers on reasonable request.

## Authors’ Contributions

J.K., T.D., T.L. and T.R. planned the study. T.D. measured soil gases, and T.R. was responsible for EIS and HPFM measuring. J.K. did statistical testing, with the exception of CLAFIC, which R.S. ran. J.K. wrote the first manuscript draft, and all the authors were involved with writing the final manuscript.

## Supplementary Material

Kilpelainen_et_al_Part1_Supporting_information_Revised_Clean_tpac092
