# Supplementary material for: Separating the effects of air and soil temperature on silver birch. Part I. Does soil temperature or resource competition determine the timing of root growth?
Source: Tree Physiol. 2022 Aug 8;42(12):2480–501. doi: 10.1093/treephys/tpac092 (PMC9743011; doi:10.1093/treephys/tpac092)
Supplement: Kilpelainen_et_al_Part1_Supporting_information_Revised_Clean_tpac092 [file kilpelainen_et_al_part1_supporting_information_revised_clean_tpac092.docx]

**Supporting information for the article “Separating the effects of air and soil temperature on silver birch. Part I. Does soil temperature or resource competition determine the timing of root growth?” by J. Kilpeläinen et al.**

**Table S1.** Total nutrient contents in soil organic layer at the beginning of the experiment (n=4).

| Element | Mean±SE |
| --- | --- |
| N (mg g^-1^) | 1.88±0.11 |
| P (µg g^-1^) | 588±20 |
| K (µg g^-1^) | 813±29 |
| B (µg g^-1^) | 1.69±0.43 |
| Ca (mg g^-1^) | 1.63±0.03 |
| Cu (µg g^-1^) | 6.98±0.40 |
| Fe (mg g^-1^) | 12.8±0.38 |
| Mg (mg g^-1^) | 2.70±0.23 |
| Mn (µg g^-1^) | 328±37 |
| S (µg g^-1^) | 256±7.1 |
| Zn (µg g^-1^) | 45.6±4.0 |

**Table S2.** Nutrient contents in long-shoot leaves of silver birch in soil temperature treatments (Cool=constant 10 °C, Warm=constant 18 °C, ECLW=Early Cool & Late Warm, EWLC=Early Warm & Late Cool) sampled 81 days after the start of the growing season (near the end of the long-day phase). Mean±SE values are shown: n=4 except for Mn in treatments Warm and ECLW n=3. The different lowercase letters indicate significant differences between treatments.

| Element | Cool | Warm | ECLW | EWLC |
| --- | --- | --- | --- | --- |
| B (µg g^-1^) | 55.9±16.7 | 57.8±7.3 | 52.6±10.1 | 42.4±15.4 |
| Ca (mg g^-1^) | 5.06±0.29 | 4.85±0.08 | 5.43±0.54 | 3.98±0.42 |
| Cu (µg g^-1^) | 3.35±0.40ab | 3.54±0.22ab | 4.77±0.57b | 3.03±0.19a |
| Fe (µg g^-1^) | 37.6±3.87a | 32.4±2.12a | 53.4±3.31b | 32.9±0.86a |
| K (mg g^-1^) | 7.76±0.75ab | 7.92±0.34ab | 10.8±1.22b | 7.04±0.47a |
| Mg (mg g^-1^) | 2.44±0.17 | 2.17±0.07 | 2.27±0.20 | 1.94±0.09 |
| Mn (µg g^-1^) | 327±38.0 | 459±30.3 | 375±23.8 | 358±39.9 |
| P (mg g^-1^) | 1.07±0.08 | 1.47±0.12 | 1.50±0.28 | 1.28±0.16 |
| S (mg g^-1^) | 1.02±0.11 | 0.89±0.06 | 1.25±0.08 | 0.88±0.09 |
| Zn (µg g^-1^) | 178±26.5 | 209±26.3 | 243±40.1 | 139±14.4 |

**Table S3.** Amounts of foliar C and nutrients of silver birch leaves in soil temperature treatments (Cool=constant 10 °C, Warm=constant 18 °C, ECLW=Early Cool & Late Warm, EWLC=Early Warm & Late Cool). Mean±SE values per tree at final harvest are shown: n=4 except for Mn in Warm treatments, and ECLW n=3. The different letters indicate significant differences between treatments. See calculation in the chapter on Soil characteristics, plant dry matter, and nutrients.

| Element | Cool | Warm | ECLW | EWLC |
| --- | --- | --- | --- | --- |
| B (mg) | 1.09±0.21ab | 2.44±0.20b | 1.27±0.36ab | 1.06±0.40a |
| C (g) | 10.5±1.70a | 20.7±1.08b | 11.6±1.70a | 12.0±0.71a |
| Ca (mg) | 108±14.9a | 208±9.91b | 130±28.3ab | 97.9±12.0a |
| Cu (ug) | 73.1±15.3a | 153±16.1b | 110±17.7ab | 74.9±6.76a |
| Fe (mg) | 0.824±0.165 | 1.40±0.140 | 1.25±0.224 | 0.812±0.057 |
| K (mg) | 175±42.3a | 340±25.8b | 244±28.3ab | 175±18.0a |
| Mg (mg) | 53.1±10.4a | 93.0±6.45b | 52.6±8.99a | 47.9±3.54a |
| Mn (mg) | 7.41±1.99a | 20.4±1.60b | 8.29±1.82a | 8.67±0.50a |
| N (mg) | 305±69.7a | 675±68.2b | 382±56.7a | 379±49.0a |
| P (mg) | 22.4±2.49a | 62.7±5.70b | 32.7±3.78a | 30.9±2.42a |
| S (mg) | 22.8±5.191 | 38.5±4.498 | 28.2±3.227 | 21.6±2.113 |
| Zn (mg) | 4.12±1.19ab | 8.92±1.17b | 5.81±1.48ab | 3.37±0.180a |

**Table S4.** Loss on ignition (LOI) and soil pH (in water) in the soil organic layer and mineral soil layers in soil temperature treatments (Cool=constant 10 °C, Warm=constant 18 °C, ECLW=Early Cool & Late Warm, EWLC= Early Warm & Late Cool) at the end of the experiment. Mean±SE values are shown: n=4. The different capital letters indicate significant differences between soil layers (*P* < 0.05). Treatment effects were not significant.

|  |  | Cool | Warm | ECLW | EWLC |
| --- | --- | --- | --- | --- | --- |
| LOI (%) | Soil organic layer | 10.9±0.60D | 11.4±0.62D | 10.0±0.42D | 12.1±0.73D |
|  | Mineral soil 0-10 cm | 3.4±0.32C | 3.2±0.46C | 2.5±0.27C | 2.5±0.20C |
|  | Mineral soil 10-20 cm | 2.0±0.05A | 1.9±0.09A | 1.8±0.07A | 1.9±0.03A |
|  | Mineral soil 20-30 cm | 2.3±0.03B | 2.3±0.05B | 2.3±0.05B | 2.3±0.09B |
| pH | Soil organic layer | 5.38±0.093A | 5.41±0.109A | 5.36±0.111A | 5.38±0.031A |
|  | Mineral soil 0-10 cm | 5.43±0.031AB | 5.51±0.085AB | 5.47±0.049AB | 5.54±0.011AB |
|  | Mineral soil 10-20 cm | 5.56±0.044BC | 5.56±0.025BC | 5.47±0.013BC | 5.65±0.053BC |
|  | Mineral soil 20-30 cm | 5.66±0.023C | 5.63±0.020C | 5.53±0.028C | 5.65±0.048B |

**Table S5.** The times (in days after the start of the first growing season (GS1)) of the initiation and cessation of short and long root elongation, and maximum root length (*l*_area_) and maximum rate of root elongation rate (Δ*l*_area_) in silver birch seedlings. GS1 was an acclimation growing season, and soil temperature treatments were present during GS2 (*n* = 4).

| Root type | Treatment | Initiation | Cessation | Max *l*_area_ | Max Δ*l*_area_ |
| --- | --- | --- | --- | --- | --- |
| Short | To be Cool in GS2 | 27±0 | 115±5 | 99±12 | 49±0 |
|  | To be Warm in GS2 | 27±0 | 120±6 | 131±17 | 49±0 |
|  | To be ECLW in GS2 | 38±6 | 125±10 | 146±15 | 49±0 |
|  | To be EWLC in GS2 | 43±5 | 125±10 | 120±24 | 49±0 |
| Long | To be Cool in GS2 | 27±0 | 110±0 | 90±12 | 38±6 |
|  | To be Warm in GS2 | 27±0 | 105±5 | 130±20 | 49±0 |
|  | To be ECLW in GS2 | 32±5 | 105±5 | 115±20 | 49±0 |
|  | To be EWLC in GS2 | 43±5 | 110±0 | 105±21 | 49±0 |

**
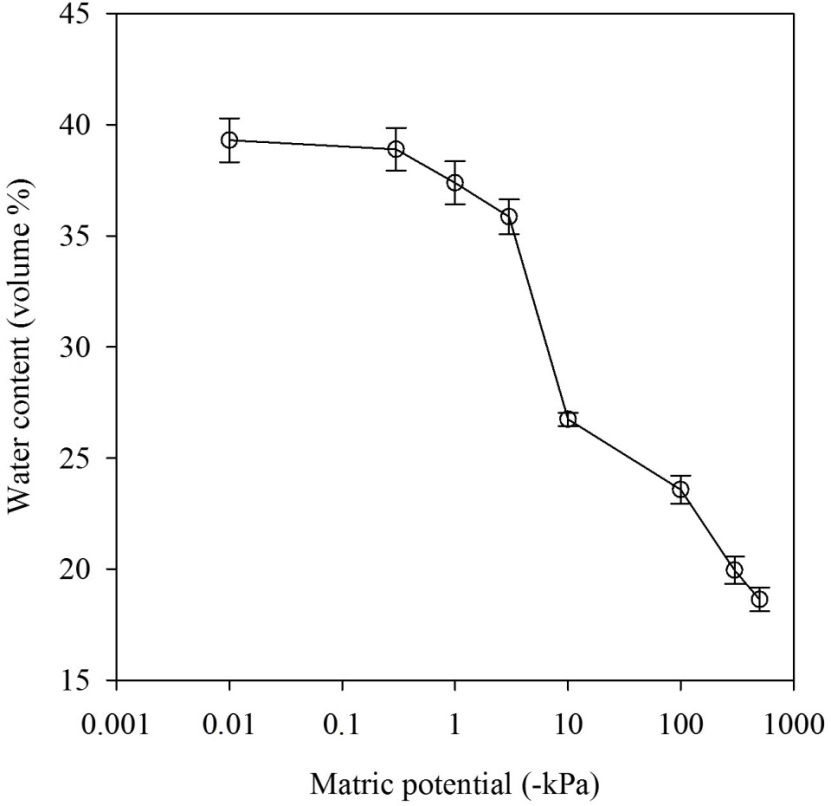
**

**Fig. S1.** Water retention curve measured for the mineral soil at desorption of (mean±SE, n=6). Mineral soil was collected in a silver birch stand for the study, and the soil was sampled in six cylinders from the growing pots, and a pressure-plate apparatus (Soilmoisture Equipment Co., Goleta, CA, USA) was used to determine the soil moisture at successive pressures (matric potentials -0.01, -0.3, -0.981, -4.905, -9.81-33, -98.1, and -500 kPa).


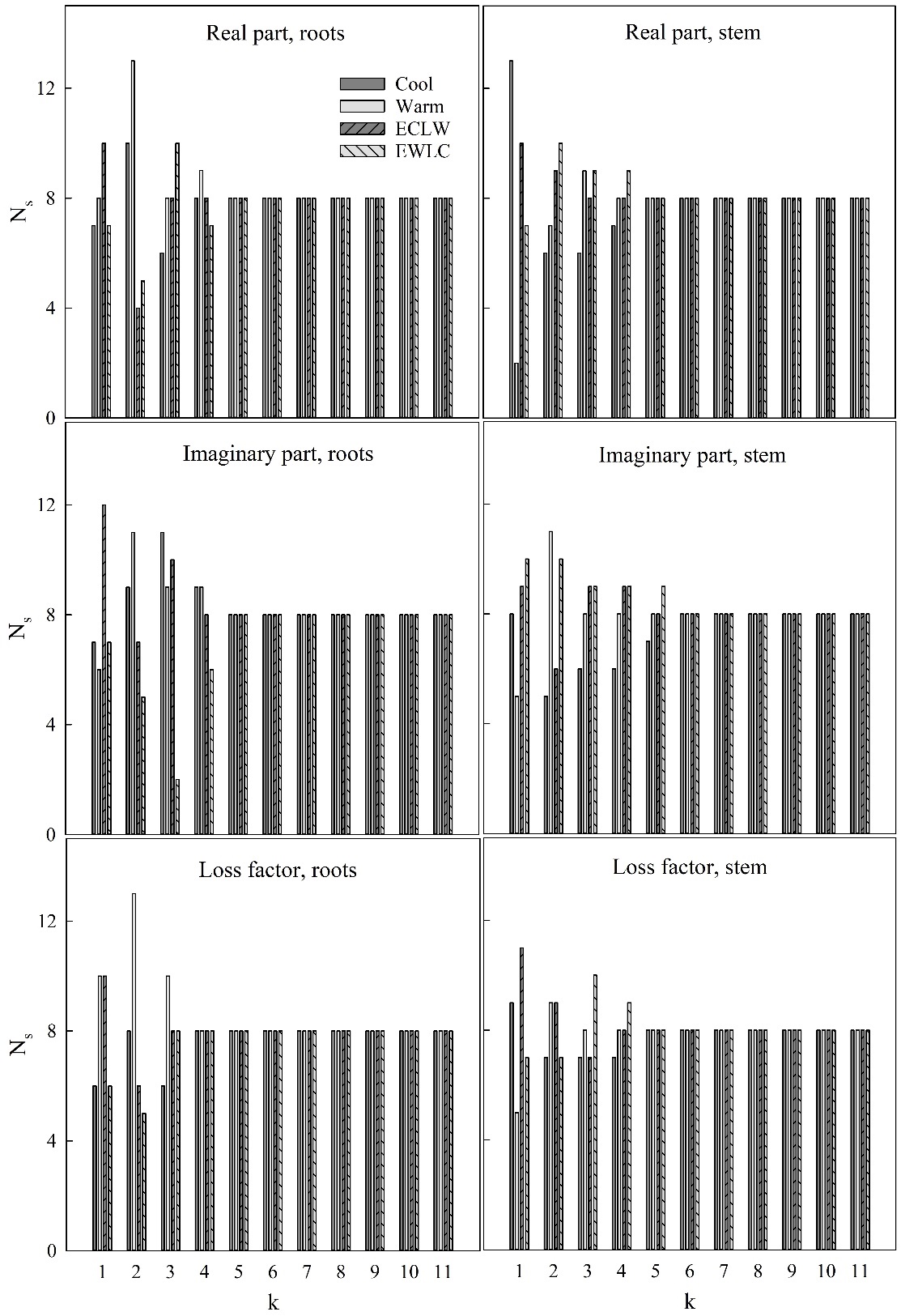


**Fig. S2.** Class-featuring information compression (CLAFIC) analysis of the real (resistance) and imaginary (reactance) parts and loss factor of the electrical impedance spectra (EIS) (data from 150 Hz to 150 kHz) for the roots and stems of the seedlings of the soil temperature treatments. N_S_ indicates the number of spectra in each classification group of Cool, Warm, ECLW, and EWLC by *k* values. Each root system and stem was measured twice.
